# NIR Photodynamic Destruction of PDAC and HNSCC Nodules Using Triple-Receptor-Targeted Photoimmuno-Nanoconjugates: Targeting Heterogeneity in Cancer

**DOI:** 10.3390/jcm9082390

**Published:** 2020-07-27

**Authors:** Shazia Bano, Girgis Obaid, Joseph W. R. Swain, Marina Yamada, Brian W. Pogue, Kenneth Wang, Tayyaba Hasan

**Affiliations:** 1 Wellman Center for Photomedicine, Massachusetts General Hospital and Harvard Medical School, Boston, MA 02114, USA; sbano@mgh.harvard.edu (S.B.); Girgis.obaid@utdallas.edu (G.O.); joewrswain@gmail.com (J.W.R.S.); yamada.m@husky.neu.edu (M.Y.); 2 Department of Bioengineering, The University of Texas at Dallas, Richardson, TX 75080, USA; 3 Department of Health Sciences, Northeastern University, Boston, MA 02115, USA; 4 Thayer School of Engineering, Dartmouth College, Hanover, New Hampshire 03755, USA; brian.w.pogue@dartmouth.edu; 5 Division of Gastroenterology and Hepatology, Mayo Clinic, Rochester, MN 55905, USA; wang.kenneth@mayo.edu; 6 Division of Health Sciences and Technology, Harvard University and Massachusetts Institute of Technology, Cambridge, MA 02139, USA

**Keywords:** intratumoral heterogeneity, biomarkers, multiple receptor-targeting, cetuximab, holo-transferrin, trastuzumab

## Abstract

Receptor heterogeneity in cancer is a major limitation of molecular targeting for cancer therapeutics. Single-receptor-targeted treatment exerts selection pressures that result in treatment escape for low-receptor-expressing tumor subpopulations. To overcome this potential for heterogeneity-driven resistance to molecular targeted photodynamic therapy (PDT), we present for the first time a triple-receptor-targeted photoimmuno-nanoconjugate (TR-PIN) platform. TR-PIN functionalization with cetuximab, holo-transferrin, and trastuzumab conferred specificity for epidermal growth factor receptor (EGFR), transferrin receptor (TfR), and human epidermal growth factor receptor 2 (HER-2), respectively. The TR-PINs exhibited up to a 24-fold improvement in cancer cell binding compared with EGFR-specific cetuximab-targeted PINs (Cet-PINs) in low-EGFR-expressing cell lines. Photodestruction using TR-PINs was significantly higher than the monotargeted Cet-PINs in heterocellular 3D in vitro models of heterogeneous pancreatic ductal adenocarcinoma (PDAC; MIA PaCa-2 cells) and heterogeneous head and neck squamous cell carcinoma (HNSCC, SCC9 cells) containing low-EGFR-expressing T47D (high TfR) or SKOV-3 (high HER-2) cells. Through their capacity for multiple tumor target recognition, TR-PINs can serve as a unique and amenable platform for the effective photodynamic eradication of diverse tumor subpopulations in heterogeneous cancers to mitigate escape for more complete and durable treatment responses.

## 1. Introduction

Photodynamic therapy (PDT) is a unique spatiotemporally controlled treatment modality that utilizes the simultaneous presence of light, a photosensitizer (PS), and oxygen. PDT is clinically approved for the treatment of various cancer and non-cancer applications [1]. Even though numerous PSs are currently approved for cancer treatment and a significant number are in clinical trials [2], the selectivity and specificity of photodamage in tumor tissues remain a challenge. PDT offers significant advantages over conventional treatment modalities in that it offers two degrees of selectivity: partial selectivity of PS tumor accumulation, and spatiotemporal selectivity inactivation. However, when PDT is applied to tumors at sensitive anatomical sites, such as pancreatic ductal adenocarcinoma (PDAC) and head and neck squamous cell carcinoma (HNSCC), there becomes a critical need for molecular precision of photodamage to preserve function, prevent potentially problematic off-target effects, and maintain aesthetics. Strategies using tumor receptor-targeting combined with PDT have opened up new possibilities to further improve tumor tissue-specific treatments.

Antibody-targeted PDT (photoimmunotherapy, PIT) is an important light-based treatment that capitalizes on antibodies specific for tumor-associated receptors [3] which are chemically coupled to PSs. These antibody-PS conjugates, also referred to as photoimmunoconjugates (PICs), have been used for PIT of cancer in vitro, in vivo, and in patients [4,5,6,7,8] for over three decades. Our prior work shows that PIT increases the specificity of tumor tissue accumulation, increases the tolerability to high doses of PDT, and significantly improves treatment outcomes [9,10,11,12,13,14]. More recently, Phase 1/2 trials (ClinicalTrials.gov Identifier: NCT02422979) of PIT using a conjugate of the silicon phthalocyanine PS derivative IRDye700DX with cetuximab (Cet, anti-EGFR) have been completed for recurrent HNSCC patients who cannot be effectively treated with chemotherapy, radiation therapy, or surgery. Findings of the clinical trials have already shown that PIT can be safe, well-tolerated, and effective in recurrent and untreatable HNSCC patients [15]. However, as with all other single-receptor-targeted therapies, PIT is not always capable of complete tumor eradication due to intratumoral receptor heterogeneity and the survival of residual resistant subpopulations.

Intratumoral receptor heterogeneity is a well-known phenomenon in most cancers and often promotes cancer progression [16,17,18,19,20]. Within a bulk tumor, distinct cellular subpopulations exist, which are often heterogeneous in their expression of cell surface receptors. This heterogeneity in receptor expression has been linked to varying degrees of resistance to treatment [21]. Single-receptor-targeted therapies generally eradicate one subpopulation while the other surviving subpopulations of tumor cells will potentially remain to proliferate and regrow in an oftentimes more aggressive fashion, promoting tumor progression. Within different subpopulations in tumors, upregulated and constitutively activated receptors, proliferation rates, and differentiation can all contribute to treatment resistance. Thus, the intratumoral response to receptor-targeted therapies can be highly variable and selection pressures exerted by these therapies often result in residual treatment-resistant tumor subpopulations. Heterogeneous over-expression of tumor-associated receptors, such as epidermal growth factor receptor (EGFR), human epidermal growth factor receptor 2 (HER-2), and transferrin receptor (TfR), has been reported in a wide range of tumors, including pancreatic ductal adenocarcinoma (PDAC) [22,23,24] and head and neck squamous cell carcinoma (HNSCC) [25,26,27,28,29,30,31,32], in addition to breast cancers [33,34,35], ovarian cancer [36,37], non-small cell lung cancer [38,39,40,41], and bladder cancer [42,43]. As such, EGFR and HER-2 in particular are well-established therapeutic targets for a variety of solid tumors [44,45,46,47,48,49,50]. For example, Cet (anti-EGFR mAb) is used for the treatment of colorectal cancer [51] and head and neck cancer [52], panitumumab (anti-EGFR mAb) has been approved for colorectal cancer [51], and necitumumab (anti-EGFR mAb) is used to treat squamous cell carcinoma of the lung [53]. Furthermore, trastuzumab (TZ, anti-HER-2 mAb) is used in the clinic for the treatment of HER-2 positive breast cancer patients [54] and gastroesophageal junction adenocarcinoma patients [55]. Targeted therapies directed towards TfR have also established this receptor as a potential target for drug delivery. Some clinical antibodies specific for TfR have shown significant anti-tumor effects [50,56]. Despite all this progress in receptor-targeted therapies, responses to single-target treatments, such as Cet, are often short-lived and patients relapse exhibiting locally recurrent and metastatic disease [57,58]. Similarly, in the case of treatment with TZ, tumors with low HER-2 expression levels often respond poorly to treatment [59,60,61].

High-payload nanoparticle formulations of anti-cancer agents are frequently utilized to enhance tumor uptake and retention, decrease systemic toxicity, and improve the therapeutic indices as compared to the free agents [62]. Molecular targeting of nanoparticles can be achieved by surface modification with targeting ligands, such as antibodies, antibody fragments, peptides, oligonucleotides, small molecules, lectins, and others [63,64]. Tumor-targeting ligands conjugated to nanoparticles can selectively bind to over-expressed cancer cell surface receptors, improve specific cancer cell binding, and result in increased cancer cell-specific uptake of therapeutics into tumor cells via receptor-mediated endocytosis [65,66,67,68].

In the context of PDT, several approaches for molecular tumor targeting employing nanoconstructs have been used for the photodynamic destruction of tumor cells over-expressing EGFR [65], HER-2 [69,70], and TfR receptors [71]. The dual- and multi-targeted approach of targeting receptors on cancer cells [67,68,72,73,74,75,76] holds significantly more potential in facilitating the specific delivery of therapeutics, such as PSs and chemotherapeutics, in vitro and in vivo, as compared with more conventional single-receptor-targeted nanoparticle approaches. High-PS payload liposomal nanoconstructs are attractive platforms for multi-specific targeted PDT due to their ability to incorporate a high number of various ligands on their surface to achieve specificity for multiple corresponding receptors [66,72,77].

Our recently published work has established a platform employing a multivariant specificity tuning approach to engineer EGFR-targeted, NIR-activatable photoimmuno-nanoconjugates (PINs) [65]. These specificity tuned PINs containing high PS payloads demonstrated up to 100-fold cancer cell binding specificities and efficient photodynamic destruction in tumor cells and nodules over-expressing cell surface EGFR. Furthermore, we showed that the molecular specificity of photodynamic destruction was also possible in a desmoplastic heterocellular in vivo model of PDAC, whereby the targeted approach was more effective at inducing tumor necrosis, and less prone to inducing off-target tissue photodamage and systemic toxicity than the untargeted controls. However, EGFR over-expression is not always homogenous in tumors, and a certain degree of cell surface HER-2 and TfR over-expression is also found in patients with PDAC [24,61] and HNSCC [27,31,78], as described earlier. Hence, the strategy can be improved by broadening the scope of molecular specificity for the PINs to increase the success of complete eradication of heterogeneous tumors. Besides, the multi-receptor-targeting approach can potentially enhance cellular binding and increase PS accumulation in cells over-expressing more than one target receptor.

In this study, we have developed NIR-activable, triple-receptor-targeted photoimmuno-nanoconjugates (TR-PINs) with three ligands, cetuximab (anti-EGFR mAb), holo-transferrin (natural ligand for TfR), and trastuzumab (anti-HER-2 mAb), conjugated to a single photosensitizing nanoconstruct to simultaneously target heterogeneous tumor cell subpopulations with differential expression levels of EGFR, TfR, and HER-2. These TR-PINs carrying a lipid-anchored derivative of the PS benzoporphyrin derivative (BPD-PC) are proposed to increase the specificity and overall completeness of PDT response in tumors with heterogeneous receptor expression. By targeting three receptors simultaneously, a diverse range of cancers from multiple tissue origins and genetic backgrounds may be effectively treated and thus resistance to monotargeted treatments that arise from receptor heterogeneity can be mitigated.

## 2. Experimental Section

### 2.1. Materials

All lipids 1-palmitoyl-2-hydroxy-sn-glycero-3-phosphocoline (16:0 Lyso PC), 1,2-Dipalmitoyl-sn-glycero-3-phosphocholine (DPPC), 1,2-dioleoyl-3-trimethylammonium-propane (chloride salt) (DOPG), cholesterol, 1,2-distearoyl-sn-glycero-3-phosphoethanolamine-*N*-[methoxy(polyethyleneglycol)-2000 (DSPE-mPEG-2000), and 1,2-distearoyl-sn-glycero-3-phosphoethanolamine-*N*-[dibenzocyclooctyl(polyethylene glycol)-2000] (ammonium salt) (DSPE-PEG2000-DBCO) were obtained from Avanti Polar Lipids, Inc. (Alabaster, AL, USA), 4-(dimethylamine) pyridine 1-ethyl-3-(3dimethylaminopropyl) carbodiimide (EDC), (DMAP), and *N*, *N*-Diisopropylethylamine (DIPEA) were from Sigma-Aldrich (St. Louis, MO, USA), Methanol, Dichloromethane (DCM, ACS Reagent Grade, 99.5%), *N*-hydroxysuccinimidyl azido poly ethylene glycol (NHS-PEG4-N3), Alexa Fluor^®^ 488 ((AF488-NHS), and Chloroform were from Fisher scientific (Waltham, MA, USA) and Verteporfin (Benzoporphyrin; BPD) was purchased from US Pharmacopeia (Rockville, MD, USA).

### 2.2. Cell Culture

Cells were cultured in Corning^®^ T75 cell culture flasks (Corning^TM^, Corning, NY, USA). A431 cells (ATCC), MIA PaCa-2 cells (ATCC), and SCC-9 cells (ATCC) were cultured in Dulbecco’s modified Eagle’s medium (DMEM). T47D cells (ATCC) were maintained in Roswell Park Memorial Institute 1640 (RPMI) medium, CHO-WT cells (kindly provided by Dr. T. Heitner at the Department of Anesthesiology, UCSF, San Francisco, CA, USA) [79] in F-12k medium (Ham’s F-12K Nutrient Mixture, Kaighn’s Mod), and SKOV-3 cells were cultured in McCoy’s 5A medium. All media were supplemented with L-glutamine, 10% heat-inactivated Fetal Bovine Serum (FBS, Gibco, ThermoFisher, Waltham, MA, USA), and 1× Penicillin/Streptomycin (Mediatech, Manassas, VA, USA). Cells were maintained in a humidified incubator at 37 °C in an atmosphere of 5% carbon dioxide, 95% air. All cells were negative for mycoplasma when tested using the MycoAlert Plus mycoplasma detection kit (Lonza, Portsmouth, NH, USA).

### 2.3. NHS-PEG_4_-N_3_ and AF488-NHS Conjugation to Cetuximab, Holo-Transferrin, and Trastuzumab

Trastuzumab ((TZ, 145,531.5 g/mol; FASTA sequence analysis); Herceptin^®^; Genentech, San Francisco, CA, USA)) and human holo-transferrin (HT, 79,680 g/mol; Sigma-Aldrich, St. Louis, MO, USA) were modified through the conjugation of *N*-hydroxysuccinimidyl azido poly-ethylene glycol (NHS-PEG4-N_3_, 88.37 g/mol; Thermo Scientific, Waltham MA, USA) to the lysine residues of the proteins, following our established protocol for the modification of Cetuximab ((Cet, 145,781.6 g/mol FASTA sequence analysis); ERBITUX^®^; Ely Lily, Indianapolis, IN, USA) [65]. Briefly, a 5-fold molar excess of NHS-PEG_4_-N_3_ in anhydrous dimethyl sulfoxide (DMSO) was reacted to the protein solution (2 mg/mL in 1× DPBS) in the presence of 2.5-fold molar excess of N-hydroxysuccinimidyl ester of Alexa Fluor^®^ 488 (AF-NHS; 643.4 g/mol, Fisher scientific, Waltham, MA, USA). The mixture was subjected to orbital rotation for 24 h at 4 °C. AF- and PEG_4-_N_3_-conjugated proteins Cet-AF-PEG_4_-N_3_, HT-AF-PEG_4_-N_3_, and TZ-AF-PEG_4_-N_3_ were purified using a pre-equilibrated (1× DPBS) PD-10 desalting column packed with Sephadex G-25 resin (GE Healthcare Life Sciences, Chicago, IL, USA) to remove any unreacted NHS-PEG_4_-N_3_ and AF-488. Protein conjugates were further concentrated in Amicon ultrafiltration tubes (30 kDa molecular weight cut off, EMD Millipore Burlington, MA, USA) by centrifuging at 2500× *g* at 4 °C. The molar concentrations (M) of the purified Cet (ε280 nm = 217,315 M^−1^cm^−1^), HT (ε280 nm = 83,360 M^−1^cm^−1^), and TZ (ε280 nm = 225,005 M^−1^cm^−1^) and the attached AF (ε494 nm = 71,000 M^−1^cm^−1^) were determined using Nanodrop One (Thermo Scientific, Waltham, MA, USA) and stored at 4 °C in dark.

### 2.4. Synthesis of Photosensitizing-Nanoconstructs (Untargeted-PSN)

Prior to liposomal preparation, Benzoporphyrin derivative (BPD) photosensitizer was anchored to the phospholipid 1-palmitoyl-2-hydroxy-sn-glycerol-3-phosphocholine (16:0 Lyso PC) through Steglich esterification [65,80]. Briefly, 16:0 Lyso PC (495.63 g/mol), BPD (718.79 g/mol), *N*-(3-dimethylaminopropyl)-*N*′-ethylcarbodiimide hydrochloride (EDC.HCl, 191.703 g/mol), 4-dimethylaminopyridine (DMAP, 122.17 g/mol), and *N,N*-diisopropylethylamine (DIPEA, 129.24 g/mol) were dissolved in dicholoromethane (DCM, 5 mL) at a 1:5:50:25:60 molar ratio, respectively, and stirred for 72 h in the dark at room temperature. The product 16:0 Lyso PC-BPD (BPD-PC) was purified on preparative thin layer chromatography (TLC) silica plates (Sigma-Aldrich, St. Louis, MO, USA) using a mobile phase of 10% methanol in DCM. Following extraction from the TLC, silica sedimentation, and removal of insoluble silica precipitates (details described previously) [65,80], the purified BPD-PC was redissolved in chloroform and stored at −20 °C in the dark.

For the preparation of liposomal photosensitizing-nanoconstructs (untargeted-PSNs), all the lipids including DPPC (734.04 g/mol), DOPG (797.02 g/mol), Cholesterol (386.65 g/mol), DSPE-mPEG-2000 (2803.79 g/mol), and DSPE-PEG2000-DBCO (3077.80 g/mol) were mixed at a ratio of 57.6:7.9:28.9:4.5:0.5 mol% with 0.6 mol% of lipidated BPD (BPD-PC) and dried to remove the chloroform under a gentle nitrogen gas flow to form a thin film. The lipid films were kept under vacuum for an additional 1 h. Dried lipid films were hydrated with 1 mL of 1× DPBS (without Ca and Mg) and were subjected to 5 freeze–thaw cycles, consisting of incubation in 45 °C water (10 min), vortexing (30 s), and incubation in ice at 4 °C (5 min). Multilamellar vesicles were then sequentially extruded through polycarbonate membranes (100 nm pore size, Avanti^®^ Polar Lipids, Inc. Alabaster, AL, USA) using a mini-extruder system (Avanti Polar Lipids, Inc. Alabaster, AL, USA)) for five extrusion cycles to prepare small unilamellar liposomes.

### 2.5. Preparation of Photoimmuno-Nanoconjugates (PIN)

All liposomal-based formulations of photoimmuno-nanoconjugates (PIN) were prepared by reacting AF-PEG_4_-N_3_-labeled proteins to untargeted photosensitizing-nanoconstructs (untargeted-PSN). Untargeted-PSNs were mixed with either Cet-AF-PEG_4_-N_3_, HT-AF-PEG_4_-N_3_, or TZ-AF-PEG_4_-N_3_ to make Cet-PINs, HT-PINs, or TZ-PINs, respectively. Cet-AF-PEG_4_-N_3_ and TZ-AF-PEG_4_-N_3_ were mixed with untargeted-PSNs to make Cet- and TZ-targeted PINs (Cet-TZ-PINs). Cet-AF-PEG_4_-N_3_, HT-AF-PEG_4_-*N*_3_, and TZ-AF-PEG_4_-N_3_ were added to untargeted-PSNs to make triple-receptor-targeted PINs (TR-PINs). The mixtures were incubated at room temperature for 24 h on rotation, using an orbital mixer to allow the copper-free click conjugation. All PINs were purified to remove unbound proteins using size exclusion columns packed with Sepharose CL-4B (Sigma-Aldrich, St. Louis, MO, USA) pre-equilibrated with 1× DPBS. Purified fractions containing protein-conjugated PINs were collected and stored in the dark at 4 °C.

### 2.6. Physical Characterizations

BPD-PC concentration (nM) within the purified conjugates of 16:0 Lyso PC-BPD or in liposomal nanoconstructs (untargeted-PSNs or PINs) was determined by diluting in DMSO and measuring the absorption spectrum using UV–visible absorption spectrophotometry (ε687 nm = 34,895 M^−1^ cm^−1^) [65,80].

The approximations of ligands (Cet, HT, TZ) attached on the surface of untargeted-PSNs were derived as described previously [65]. Fluorescence emission (Exc = 480 nm, Emi = 517 nm) of all purified PINs and BPD-PC concentration (nM) within each PINs were used to derive the conjugation efficiency (%) of the ligand (Cet, HT, TZ) to the untargeted-PSN.

Untargeted-PSNs and PINs were characterized with regards to their hydrodynamic diameter (nm), polydispersity index (PDI), and ς-potential (mV) using the Zetasizer Nano ZS Dynamic Light Scattering Instrument (Malvern Instruments, Ltd., Houston, TX, USA). Measurements were performed in triplicates and values were reported as mean and standard deviation.

### 2.7. Singlet Oxygen Measurements

Singlet oxygen measurements were performed in a 96-well plate (black wall, transparent bottom) using Singlet Oxygen Sensor Green (SOSG) (Thermo Fisher Scientific, Waltham, MA, USA) and diethyl-3-30-(9,10-anthracenediyl) bis Acrylate (DADB; a kind gift from Dr. David Kessel at Wayne State University) [80]. Briefly, solutions of untargeted-PSNs or PINs in 1× DPBS were mixed with SOSG (50 uM) or DADB (10 uM). The solutions were then irradiated at 150 mW cm^−2^ (690 nm laser) with varying fluences of 0, 5, 10, 20, 40, 60, 80, and 100 J cm^−2^. The fluorescence intensity for SOSG (Exc _460 nm_, cut-off filter _515 nm_, Emi _525 nm_) and DADB (Exc _405 nm_, cut-off filter _475 nm_, Emi _505 nm_) was measured using a Microplate Reader (Spectra Max M Series Multi-Mode) following each light dose delivery. The relative rate of ^1^O_2_ production with PINs as compared with untargeted-PSNs was calculated as

(1) $$\mathrm{Relative}\;\mathrm{rate}=\frac{\mathrm{rate}\;\mathrm{of}\;\mathrm{DADB}\;\mathrm{fluoresecence}\;\mathrm{decay}\;\mathrm{when}\;\mathrm{irradiated}\;\mathrm{in}\;\mathrm{the}\;\mathrm{presence}\;\mathrm{of}\;\mathrm{untargeted}\;\mathrm{PSNs}}{\;\mathrm{rate}\;\mathrm{of}\;\mathrm{DADB}\;\mathrm{fluoresecence}\;\mathrm{decay}\;\mathrm{when}\;\mathrm{irradiated}\;\mathrm{in}\;\mathrm{the}\;\mathrm{presence}\;\mathrm{of}\;\mathrm{PINs}}$$

### 2.8. Cellular Binding of PINs

Single-cell suspensions of 50,000 cells/microcentrifuge tubes were incubated with 250 nM BPD-PC equivalent of untargeted-PSN or PINs formulations in the respective serum-containing culture media at 37 °C for 30 min in the dark. For the approximation of expression levels of EGFR, TfR, and HER-2, MIA PaCa-2 and SCC-9 cells were incubated with 10 μg/mL of AF-conjugated proteins (Cet-AF, HT-AF, or TZ-AF) in the respective serum-containing culture media at 37 °C for 30 min in the dark.

Following incubation, the cells were centrifuged at 1000× *g* for 5 min and the supernatant was removed. Cell pellets were resuspended in 200 μL of pre-cooled 1× DPBS, agitated 5 times with a pipette to form single-cell suspensions, and transferred to flow cytometry tubes. The fluorescence intensity of cell-associated BPD-PC and Alexa Fluor 488 was measured using the BD FACSAriaTM II flow cytometer (BD Biosciences^®^, Woburn, MA, USA). Ten thousand events were recorded and gated for each group using a 405 nm laser and a 610 nm dichroic long-pass filter for BPD and a 450/40 nm filter for AL488. Median BPD-PC emission was quantified using the FlowJo^®^ software (V10, Franklin Lakes, NJ, USA). Data are presented as mean ± SEM from three biological replicates for each group. Fold improvement in binding with targeting is defined as the cellular binding of a targeted nanoconstruct with respect to the cellular binding of untargeted nanoconstructs and is calculated as

(2) $$\mathrm{Fold}\;\mathrm{improvement}=\frac{\;\mathrm{Cellular}\;\mathrm{binding}\;\mathrm{of}\;\mathrm{PINs}}{\mathrm{Cellular}\;\mathrm{binding}\;\mathrm{of}\;\mathrm{untargeted}\;\mathrm{PSNs}}$$

### 2.9. In vitro PINs Internalization Studies

MIA PaCa-2 and SCC-9 cells at 70–90% confluence were seeded in 24-well, glass-bottom, black-walled plates at a density of 1 × 10^5^ cells/well. Adherent cells were then incubated for 6 h with Cet-PINs, Cet-TZ-PINs, or TR-PINs formulations at 250 nM BPD-PC equivalent concentration in the respective serum-containing cell media and kept in the dark at 37 °C. Prior to imaging, cells were washed twice with 1× DPBS and were stained with 50 nM LysoTracker^®^ Red DND-99 (Invitrogen, Carlsbad, CA, USA) at 37 °C in the dark. Hoechst^®^ 33,342 (Invitrogen, Carlsbad, CA, USA) was used to stain the nuclei of the cells prior to fluorescence imaging. Images were acquired using a confocal microscope (Olympus FluoView-1000 confocal microscope) through a 60× objective (1.2NA, Water). The nuclei, lysosomes, and BPD-PC were visualized using 405 (Hoechst and BPD) and 559 nm (LysoTracker) laser excitation, respectively, with appropriate filters (Hoechst: 425–475 nm; LysoTracker: 580–650 nm; BPD-PC: 655–755 nm).

Flow cytometry was also used for the quantification of intracellular uptake after 6h incubations. Cells were seeded in 24-well, glass-bottom plates at a density of 2 × 10^5^ cells/well, incubated with untargeted-PSN or PINs formulations at 250 nM BPD-PC equivalent concentration in media for 6 h, washed twice in 1× DPBS harvested with trypsin, and transferred to flow cytometry tubes following subsequent washing with 1× DPBS and pipette agitation as described before. Ten thousand events were recorded and gated for each group. BPD-PC emission was quantified using the FlowJo^®^ software (V10, Franklin Lakes, NJ, USA). Data are presented as mean ± SEM from three biological replicates for each group. An increase in cellular uptake of PINs as compared with untargeted-PSN was calculated as

(3) $$\mathrm{Fold}\;\mathrm{increase}\;\mathrm{in}\;\mathrm{cellular}\;\mathrm{uptake}=\frac{\;\mathrm{Cellular}\;\mathrm{uptake}\;\mathrm{of}\;\mathrm{PINs}}{\mathrm{Cellular}\;\mathrm{uptake}\;\mathrm{of}\;\mathrm{untargeted}\;\mathrm{PSNs}}$$

### 2.10. Photodynamic Treatment of PDAC and HNSCC Monocellular and Heterocellular 3D Nodules and Image Analysis

Suspended 3D nodules of MIA PaCa-2 and SCC-9 cells were grown and cultured in 96-well, black-walled, round-bottom ultralow attachment plates (Corning^®^ Costar^®^, Corning, NY, USA) at 37 °C. MIA PaCa-2 cells were seeded at a density of 2.5 × 10^3^ cells per well and SCC-9 cells were seeded at a density of 5 x 10^3^ cells per well for 48 h to self-assemble into single 3D nodules. Nodules were then incubated with untargeted-PSN or PINs formulations at varying concentrations of BPD-PC. After 6h of incubation, nodules were washed three times with 100ul of the respective serum-containing cellular media and irradiated with 40 J/cm^2^ of 690 nm laser light (Intense, North Brunswick, NJ, USA) at an irradiance of 150 mW/cm^2^. At 72 h following photodynamic activation, cells were co-stained with LIVE (Calcein AM; Invitrogen, Carlsbad, CA, USA) and DEAD (propidium iodide) reagents to analyze the viability of treated cells. Prior to staining, nodules for total killing control were fixed using a 10% formalin solution in 1× DPBS (2–4 min) and cell membranes were permeabilized with 0.1% Triton X-100 incubation (60 min) and washed with 0.1 M Glycine (3 times). Nodules were then incubated with calcein AM (Invitrogen, Carlsbad, CA, USA) and Propidium Iodide (Sigma-Aldrich, St. Louis, MO, USA) at standard culture conditions according to the manufacturer’s protocol.

Fluorescence signals were recorded using an Olympus FV-1000 confocal microscope through a 0.16NA 4x air objective at λexc = 488 nm/λem = 520 nm (calcein) and λex = 559 nm/λem = 630 nm (PI). Brightfield images were acquired under 559 nm light. The acquisition was standardized for each nodule. All experimental conditions were performed with an *n* of 8–12 nodules. Comprehensive high-throughput image analysis (CALYPSO) was used to generate heat map images and for quantifying the fractional viability [81].

## 3. Results

### 3.1. Design, Preparation, and Characterization of Photoimmuno-Nanoconjugates (PINs)

Untargeted-photosensitizing-nanoconstructs (PSNs) were prepared from anionic DOPG-containing DPPC liposomes and a lipid-anchored derivative of benzoporphyrin derivative (BPD-PC), as described previously [65,80]. The anionic charge is required to minimize the variability in uptake between multiple cell lines [65]. The untargeted-PSNs also contained DSPE-PEG_2000_ with a dibenzocyclooctyle (DBCO) functional group to further allow for the covalent conjugation of the targeting ligands through copper-free click chemistry. Liposomal nanoconstructs hold great promise as drug delivery vehicles for emerging treatment regimens due to their ability to carry multiple payloads that can be tuned with regard to their hydrophilicity or hydrophobicity. Furthermore, their ability to incorporate multiple surface-targeting ligands of varying natures with finely tunable surface densities is a particularly important attribute required for precision medicine.

**Figure 1:**
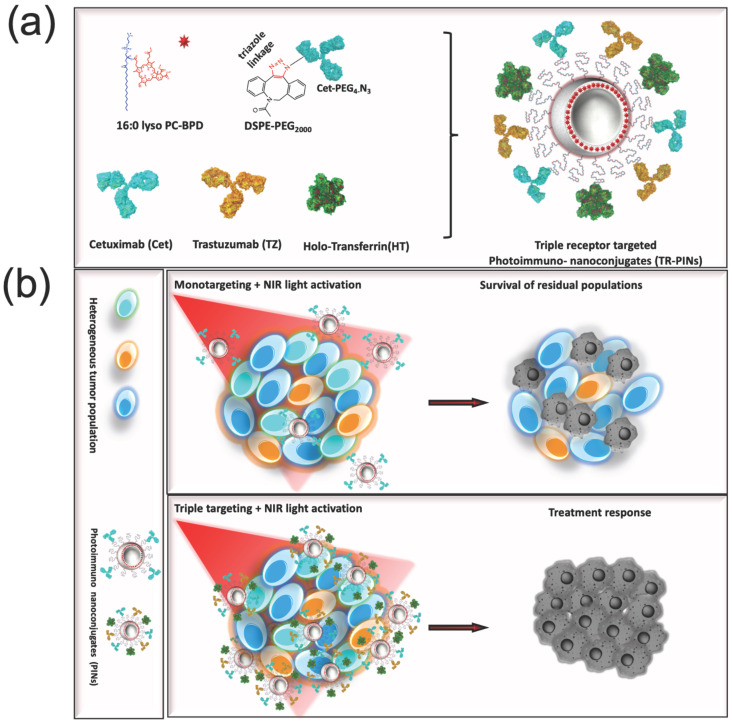
Schematic of the design and selective targeting of heterogeneous cancer cell subpopulations using triple-receptor-targeted photoimmuno-nanoconjugates (TR-PINs). Representation of (**a**) the design of triple-receptor-targeted PINs (TR-PINs). (**b**) Selective tumor photodestruction using Cet-targeted PINs (Cet-PINs) leading to incomplete responses, and enhanced specificity tumor photodestruction using TR-PINs leading to a more complete response within the heterogeneous tumor mass.

The conjugation efficiency of the individual ligands bound to the surface of the untargeted-PSNs was quantified by labeling cetuximab (Cet) with Alexa Fluor 488, holo-transferrin (HT) with Alexa Fluor 647, and trastuzumab (TZ) with Alexa Fluor 680. Untargeted-PSNs and photoimmuno-nanoconjugates (PINs), including HT-targeted PINs (HT-PINs), TZ-targeted PINs (TZ-PINs), Cet-targeted PINs (Cet-PINs), both Cet- and TZ-targeted PINs (Cet-TZ-PINs), and triple-receptor-targeted PINs (TR-PINs) (Figure 1), exhibit an average hydrodynamic size of 130.57 ± 9.2, and polydispersity index (PDI) of 0.06 ± 0.01, which is suggestive of a narrow size distribution and monodisperse nanoconstructs. The constructs all exhibited a ζ-potential between −16.7 and −18.6 mV, demonstrating that an anionic charge is maintained in all PINs prepared (Table 1). A consistent ζ-potential is important for minimizing variability in uptake that is not associated with the nature of the targeting ligand or ligands.

### 3.2. Cellular Binding Specificity of Photoimmuno-Nanoconjugates (PINs)

**Figure 2:**
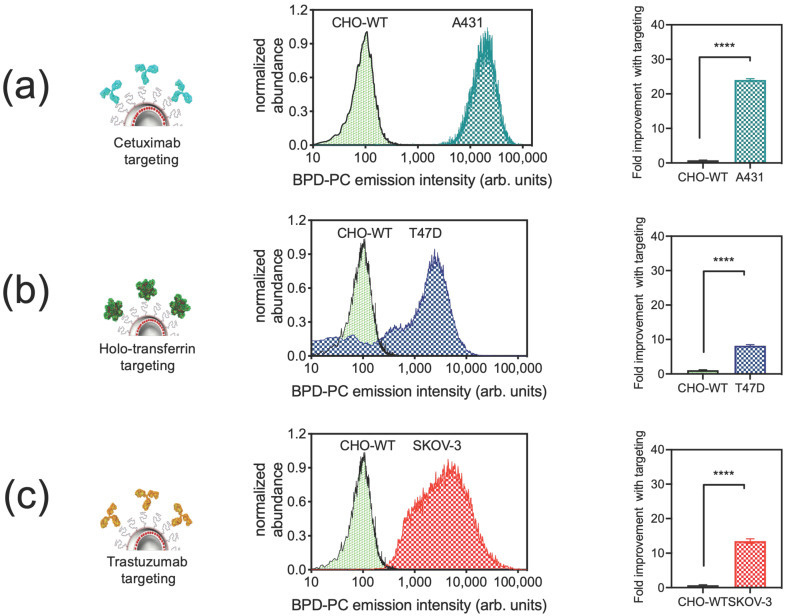
Cellular binding specificity of photoimmuno-nanoconjugates (PINs) to tumor cells over-expressing EGFR, TfR, and HER-2 receptors. Flow cytometry histograms and bar graphs representing the specificity of PINs conjugated to the tumor-specific ligands cetuximab (to target EGFR), holo-transferrin (to target TfR), or trastuzumab (to target HER-2). Binding specificity of (**a**) Cet-PINs to A431 cells (high EGFR), (**b**) HT-PINs to T47D cells (high TfR), and (**c**) TZ-PINs to SKOV-3 cells (high HER-2) is presented with respect to the untargeted-PSNs for each cancer cell line and the control CHO-WT cell line (null for EGFR, TfR, HER-2). (mean ± S.E.M.; unpaired *t*-test, n = 3 for each cell line; **** = *p* ≤ 0.0001).

We have recently shown for the first time that our chemically tuned NIR light-activated Cet-PINs targeted to a single receptor, EGFR, selectively binding, permeating, and destroying tumor cells in a 3D heterocellular pancreatic ductal adenocarcinoma (PDAC) model more efficiently than untargeted-PSNs [65]. In this study, we have further modified the design of Cet-PINs to direct the construct towards additional tumor-associated receptors (HER-2 and TfR) that are over-expressed in several cancers including PDAC and HNSC. The cellular binding was measured by the quantitation of the BPD-PC fluorescence intensity from the nanoconstructs. A431 (high EGFR) [82], T47D (high TfR) [83,84], SKOV-3 (high HER-2) [85,86], and CHO-WT (EGFR null) [87] cells were incubated with untargeted-PSNs or targeted PINs (Cet-PINs, HT-PINs, TZ-PINs, Cet-TZ-PINs, TR-PINs) to determine the cellular binding specificity using flow cytometry. Cet-PINs, HT-PINs, and TZ-PINs exhibit higher cellular association in high-receptor-expressing cancer cells than the untargeted-PSNs (Figure 2).

As is consistent with our previous findings [65], Cet-PINs improved nanoconstruct binding to A431 cells (high EGFR) by 24-fold (Figure 2a), as compared with untargeted-PSNs. Although elegant prior work has shown that liposomal Foscan^®^ targeted with transferrin exhibited no cellular specificity [71], our HT-PINs demonstrated an 8-fold improvement in T47D cell (high TfR) binding, as compared with untargeted-PSNs (Figure 2b). This discrepancy with the prior work is most likely due to the nanoconstruct membrane-stabilizing effect that lipid anchoring of BPD has in our studies, that prevents the non-specific transfer of the photosensitizer when the construct is not targeted [65,80]. TZ targeting also improved the binding of TZ-PINs to SKOV-3 cells (high HER-2) by 13.5-fold (Figure 2c). As expected, no significant binding of Cet-PINs, HT-PINs, or TZ-PINs to CHO-WT cells was observed due to the absence of expression of all three receptors [87,88] (Figure 2a–c).

**Figure 3:**
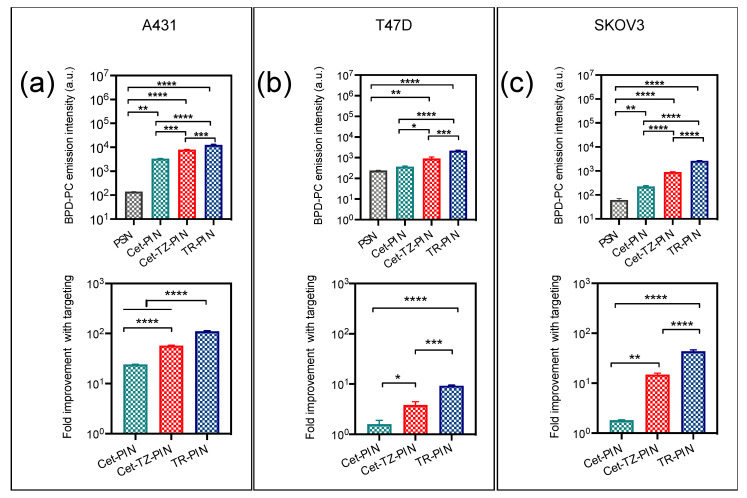
Triple-receptor targeting enhances the TR-PIN binding to cells expressing corresponding receptors. Cellular binding and corresponding BPD-PC emission intensities of PINs using flow cytometry analysis. Triple-receptor-targeted PINs (TR-PINs) exhibiting a significant improvement in binding with TR-PINs to (**a**) A431 cells, (**b**) T47D cells, and (**c**) to SKOV-3 cells in comparison with Cet-targeted PINs (Cet-PINs) or Cet- and TZ-targeted PINs (Cet-TZ-PINs) (mean ± S.E.M.; n = 3; one-way ANOVA with a Tukey post-test; **** = *p* ≤ 0.0001; *** = *p* ≤ 0.001; ** = *p* ≤ 0.01; * = *p* ≤ 0.05).

Cellular binding of Cet-PINs to A431 cells (24-fold improvement with targeting) was higher as these cells have a higher EGFR expression (2–4 × 10^6^ EGFR/cell) [82] than T47D (7 × 10^3^ EGFR/cell) [89] or SKOV-3 cells (6.3 × 10^4^ EGFR/cell) [90]. As expected, binding of Cet-PINs in T47D and SKOV-3 cells which express low levels of EGFR was only improved by 1.5- and 1.8-fold, respectively, with targeting as compared with untargeted-PSN controls (Figure 3).

Cellular binding of Cet-PINs to A431 cells (24-fold improvement with targeting) was higher as these cells have a higher EGFR expression (2–4 × 10^6^ EGFR/cell) [82] than T47D (7 × 10^3^ EGFR/cell) [89] or SKOV-3 cells (6.3 × 10^4^ EGFR/cell) [90]. As expected, binding of Cet-PINs in T47D and SKOV-3 cells which express low levels of EGFR was only improved by 1.5- and 1.8-fold, respectively, with targeting as compared with untargeted-PSN controls (Figure 3).

Cet-TZ-PINs exhibited marked improvements in cellular binding (Figure 3), as compared with EGFR targeting alone, providing dual specificity in A431 cells (57.1-fold improvement with targeting) due to the presence of higher HER-2 receptors (1–2 × 10^5^ HER-2/cell) [91]. As SKOV-3 cells have elevated levels of HER-2 (1.6 × 10^6^ HER-2/cell) [86], the dual targeting specificity (19.1-fold improvement with targeting) was lower than in A431 cells because of the low EGFR (6 × 10^3^ EGFR/cell) expression levels in SKOV-3 cells (Table 2). Binding to T47D cells was only improved 3.8-fold with targeting when compared with the untargeted-PSN, due to fact that both EGFR (7 × 10^3^ EGFR/cell) and HER-2 (3 × 10^4^ HER-2/cell) expression levels are low in that cell line [89] (Figure 3b).

The TR-PINs enhanced the cellular binding, providing significant improvements of up to 111-fold, 43.6-fold, and 9.2-fold binding in A431 cells (Figure 3a), SKOV-3 cells (Figure 3c), and T47D cells (Figure 3b), respectively, as compared with untargeted-PSNs. The binding of TR-PINs was highest in A431 cells due to its high over-expression of all three receptors (2–4 × 10^6^ EGFR/cell [82], 1.2 × 10^5^ TfR/cell [92], and 1–2 × 10^5^ HER-2/cell) [91]. The importance of these findings is that they emphasize how the amenability of the PIN platform can be leveraged to modulate multispecificity that ultimately targets heterogeneous tumor cell populations, in addition to increasing the receptor-specific uptake of PS-containing nanoconstructs in cancer cells.

### 3.3. Triple Receptor Targeting Enhances PIN Binding and Cellular Uptake in MIA PaCa-2 PDAC Cells and SCC-9 HNSCC Cells

We hypothesize that heterogeneous tumors such as PDAC and HNSCC exhibiting diverse patterns of tumor-associated cell surface receptors (EGFR, TfR, HER-2) over-expression, can be selectively targeted using PDT directed against EGFR, TfR, and HER-2 concurrently. TR-PINs would enable the specific recognition of multiple cell surface targets and would increase the specificity of drug delivery and treatment efficacy in heterogeneous tumor environments, thereby ultimately mitigating treatment escape.

**Figure 4:**
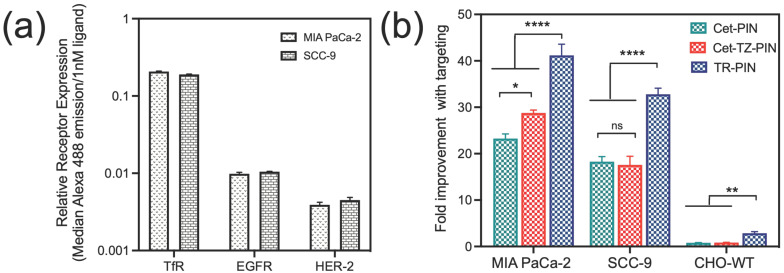
Triple-receptor-targeted PINs (TR-PINs) achieve higher binding specificity in MIA PaCa-2 and SCC-9 cells. (**a**) Relative receptor expression of EGFR, HER-2, and TfR in MIA PaCa-2 and SCC-9 cells determined using flow cytometry and represented as median fluorescence emission of Alexa Fluor 488/1nM ligand, when conjugated to cetuximab, transferrin, or trastuzumab, respectively. (**b**) Cellular binding data using Cet-targeted PINs (Cet-PINs), Cet- and TZ-targeted PINs (Cet-TZ-PINs), and triple-receptor-targeted PINs (TR-PINs) show a significant improvement in binding with TR-PINs, as compared with Cet-PINs or Cet-TZ-PINs in MIA Paca-2 and SCC-9 cells. No obvious change in cellular binding in CHO-WT cells was observed with TR-PINs due to the lack of receptor expression. (mean ± S.E.M.; n = 3–9; one-way ANOVA with a Tukey post-test; **** = *p* ≤ 0.0001; ** = *p* ≤ 0.01; * = *p* ≤ 0.05).

Relative cell surface expression levels of EGFR, TfR, and HER-2 in MIA PaCa-2, SCC-9 (Figure 4a) and SKOV-3 cells (Figure S1) were determined using flow cytometry data. The median emission intensities (a.u) of the individual ligands (conjugated AF-488) when bound to the cells were corrected for differences in the brightness of the individual ligand conjugates by normalizing to their respective fluorescence intensities (a.u)/1 nM ligand. This provided relative expression levels of the corresponding receptors expressed in each cell line. Using the established EGFR expression levels in MIA PaCa-2 cells (1.7 × 10^5^ EGFR/cell) [93], we approximate that MIA PaCa-2 cells express 3.5 × 10^6^ TfR/cell and 6.7 × 10^4^ HER-2/cell. Similarly, in SCC-9, we approximate that SCC-9 cells express 1.8 × 10^5^/EGFR-2/cell, 3.2 × 10^6^ TfR/cell, and 0.7 × 10^5^ HER-2/cell (Table 2).

The advantages of triple-receptor targeting are not limited to only an enhanced diversity of cancer cell surface binding (Table 3). This strategy also significantly increases the ability of PDAC and HNSCC tumor cells to internalize TR-PINs in vitro. It was found that the simultaneous targeting of EGFR, HER-2, and TfR receptors demonstrate significantly higher cellular binding of TR-PINs (Figure 4b), relative to the EGFR, TfR, and HER-2 over-expression in MIA PaCa-2 and SCC-9 cells. Triple-receptor targeting resulted in 41-fold (MIA PaCa-2 cells) and 33-fold (SCC-9 cells) improvements in binding with targeting when compared with the untargeted-PSNs. Furthermore, a 77% (MIA PaCa-2) and 80% (SCC-9) increase in binding was observed with TR-PINs in comparison with Cet-PINs (Figure 4b). As expected, no notable changes in cellular binding in CHO-WT cells were observed using TR-PINs due to the lack of expression of all three receptors (Figure 4b).

**Figure 5:**
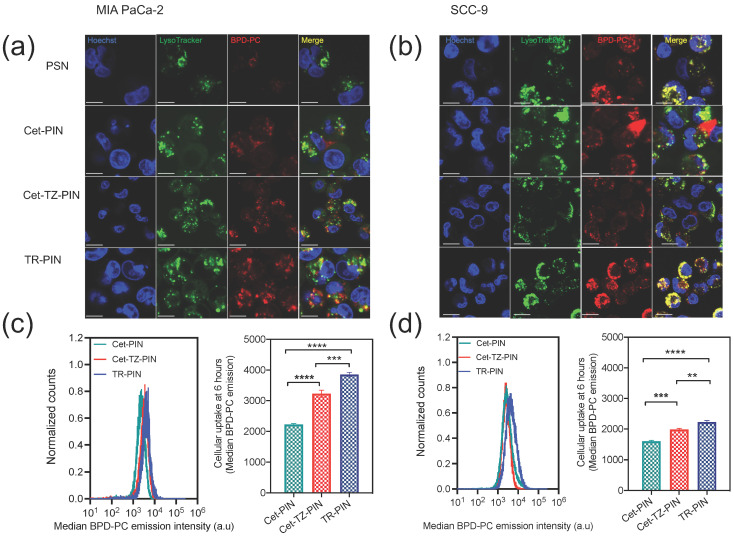
Triple-receptor targeting enhances TR-PIN cellular uptake to MIA PaCa-2 and SCC-9 cells. Confocal images (60X) demonstrate intracellular uptake of Cet-targeted PINs (Cet-PINs), Cet- and TZ-targeted PINs (Cet-TZ-PINs), and triple-receptor-targeted PINs (TR-PINs) in MIA PaCa-2 (**a**) and SCC-9 (**b**) cells after 6 h of incubation. PINs localize to lysosomal compartments as shown in the merged channel images where yellow indicates co-localization of BPD-PC (red) and lysosomes (green). The nuclei, lysosomes, and BPD-PC were visualized using 405 (Hoechst and BPD-PC) and 559 nm (LysoTracker) laser excitation. Scale bar = 50 μm. Flow cytometry quantitation of cellular uptake of PINs (median BPD-PC emission signals) is shown for **(c)** MIA Paca-2 and (**d**) SCC-9 cells at 6 h incubation. (mean ± S.E.M.; n = 6–9 for c–d; one-way ANOVA with a Tukey post-test; **** = *p* ≤ 0.0001; *** = *p* ≤ 0.001; ** = *p ≤* 0.01).

The subcellular localization of PINs in MIA PaCa-2 and SCC-9 cells was observed using confocal microscopy. Cells were incubated with PINs and nuclei and lysosomes were stained after 6 h of incubation. All PINs were found to localize to endo-lysosomal compartments, exhibiting punctate intracellular BPD-PC signals in MIA PaCa-2 (Figure 5a) and SCC-9 cells, respectively (Figure 5b). This is consistent with our previous findings for BPD-PC nanoconstructs [65,94].

Intracellular uptake of PINs was quantified using flow cytometry following 6 h of incubation with MIA PaCa-2 and SCC-9 cells. The trend in the uptake levels at 6 h incubation correspond to the cellular binding of PINs and demonstrate a significant increase in uptake of TR-PINs in MIA PaCa-2 and SCC-9 cells, compared with Cet-PINs and Cet-TZ-PINs. Quantitation of BPD-PC fluorescence intensities using flow cytometry demonstrate a 1.5-fold (45%) and 1.7-fold (73%) increase in cellular uptake of Cet-TZ-PINs and TR-PINs, respectively, in MIA PaCa-2 cells, as compared with Cet-PINs. Further, a 1.2-fold (24%) and 1.4-fold (39%) increase in cellular uptake of DR-PINs and TR-PINs, respectively, was observed in SCC-9 as compared with Cet-PINs. These results suggest that triple targeting enables the TR-PINs to bind and internalize in MIA PaCa-2 and SCC-9 cells more efficiently with respect to Cet-PINs, delivering higher levels of intracellular BPD-PC for molecular-targeted PDT.

### 3.4. Singlet Oxygen Measurements

As the PINs are nanosystem-designed for effective PDT-mediated killing, they must retain their ability to generate cytotoxic reactive molecular species, such as singlet oxygen (^1^O_2_), when functionalized with various targeting ligands. ^1^O_2_ is the predominant cytotoxic molecular species produced during the photosensitization of BPD and its lipid-anchored derivatives [1,80].

To monitor photogenerated ^1^O_2_ from NIR-activated PINs, two ^1^O_2_ probes, SOSG and DADB, were used in this study. While oxidation of SOSG with ^1^O_2_ increases the probe’s fluorescence, the endoperoxide photooxidation product of DADB is non-fluorescent and exhibits a decay in the fluorescence intensity upon reaction with ^1^O_2_. Further, DADB is lipophilic and partitions in the phospholipid bilayer of BPD-PC nanoconstructs, probing the immediate production of ^1^O_2_ [80,95], whereas SOSG is a membrane-impermeable probe that measures global ^1^O_2_ in the entire solution. We have confirmed in our previous study that BPD-PC fluorescence is negligible at the wavelengths used to monitor DADB (505 nm) and SOSG (525 nm) emission signals, confirming that both probes are appropriate for measuring ^1^O_2_ production from BPD-PC nanoconstructs [80].

**Figure 6:**
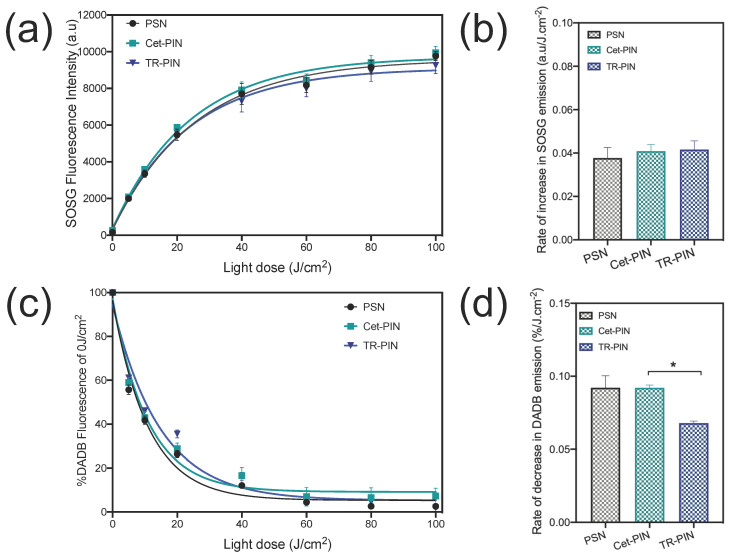
Singlet oxygen production monitored using SOSG and DADB. Singlet oxygen production as monitored using (**a**–**b**) SOSG (Exc = 460 nm, cut-off filter = 515 nm, Emi = 525 nm) and (**c**–**d**) DADB (Exc = 405 nm, cut-off filter = 475 nm, Emi = 505 nm) emission with increasing fluences of 690 nm light irradiation of untargeted-PSN, Cet-targeted PINs (Cet-PINs), and triple-receptor-targeted PINs (TR-PINs). The irradiance was maintained at 150 mW/cm^2^ throughout. (mean ± S.E.M.; n = 4–8; one-way ANOVA with a Tukey post-test; * = *p* ≤ 0.05).

Figure 6a shows a light dose-dependent increase in the SOSG fluorescence intensity following laser light irradiation. Fluorescence intensity of the SOSG in the mixture (PINs + SOSG) increased with increasing light doses (0 J/cm^2^–100 J/cm^2^), representing the generation of ^1^O_2_ and conversion of SOSG to its photo-oxidized product. No difference in the rate of photogenerated ^1^O_2_ (as measured by increased emission of SOSG) was observed following the irradiation of the untargeted-PSNs, Cet-PINs, and TR-PINs, (Figure 6a) suggesting that the global average of ^1^O_2_ in the solution is unaltered by the degree of ligand functionalization. However, when ^1^O_2_ generation was measured only in the hydrophobic membrane compartments of the PSNs and PINs using DADB (Figure 6c), differences were observed. The relative rate of ^1^O_2_ production was calculated using the equation described in Section 2.7. A 1.5-fold higher rate of ^1^O_2_ production (DADB fluorescence decay (0.09%/J cm^−2^)) was observed with both the untargeted-PSNs and Cet-PINs, as compared with the TR-PINs (0.06%/J cm^−2^) (Figure 6d). The TR-PINs have a total of 89.6 ligands per construct, whereas the Cet-PINs have a total of 27.1 ligands per construct. Given that ^1^O_2_ also reacts with aromatic amino acids that are abundant in the surface-bound targeting ligands [96], this increased number of membrane surface ligands from Cet-PINs to TR-PINs might explain the 1.5-fold reduction in the rate of ^1^O_2_ production in the TR-PIN membrane, as determined by the DADB measurements.

### 3.5. NIR Light-Mediated Photodynamic Treatment of PDAC and HNSCC Monocellular and Heterocellular 3D Models of Heterogeneity

As shown earlier, the TR-PINs exhibit expanded cancer cell binding specificities and enhanced cellular uptake in MIA PaCa-2 and SCC-9 cells and have the potential for simultaneously targeting heterogeneous tumor subpopulations in PDAC and HNSCC. As such, we further evaluated the NIR phototoxicity of the TR-PINs in PDAC (MIA PaCa-2) and HNSCC (SCC-9) 3D nodules with varying cell surface receptor expression levels of EGFR, TfR, and HER-2. Considering that EGFR over-expression is prevalent in PDAC and HNSCC, we compared the NIR phototoxicity of Cet-PINs with TR-PINs (specific for the additional receptors HER-2 and TfR) in the MIA PaCa-2 and SCC-9 3D nodules. Treatment efficacy was also evaluated in T47D and SKOV-3 3D nodules as a control for low-EGFR-expressing cells. Furthermore, T47D and SKOV-3 cells (low EGFR) were included in PDAC (MIA PaCa-2) and HNSCC (SCC-9) 3D nodules to recapitulate heterogeneous tumor cell subpopulations that would evade EGFR-targeted Cet-PINs. Targeted PDT efficacy was also evaluated in the heterocellular 3D models of heterogeneity.

**Figure 7:**
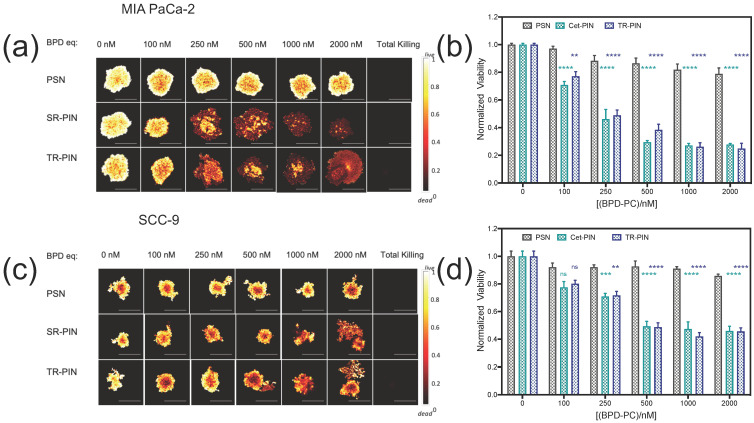
NIR light-mediated photodynamic treatment of 3D monocultures of MIA PaCa-2 and SCC-9 cells. Viability heatmap images of (**a**) 3D MIA-PaCa-2 and **(c)** SCC9 nodules following photodynamic therapy (PDT) with increasing concentrations of BPD-PC equivalent in untargeted-PSNs, Cet-targeted PINs (Cet-PINs), and triple-receptor-targeted PINs (TR-PINs) (690 nm, 40 J/cm^2^ at 150 mW/cm^2^). The comprehensive image analysis procedure for structurally complex organotypic cultures (CALYPSO) image analysis framework was used for quantitation of normalized viability following PDT (**b**,**d**). (mean ± S.E.M.; n = 8–12; one-way ANOVA with a Tukey post-test; **** = *p* ≤ 0.0001; *** *= p* ≤ 0.001; ** = *p* ≤ 0.01).

Firstly, the NIR phototoxicity of Cet-PINs and TR-PINs was assessed in monocellular 3D nodules of MIA PaCa-2 and SCC-9. The nodules were incubated for 6 h with untargeted-PSNs, Cet-PINs, or TR-PINs at 0–2000 nM BPD-PC equivalent, 48 h after seeding in round-bottom ultralow attachment plates as described in the Experimental Section. The nodules were then irradiated with 40 J/cm^2^ of 690 nm laser light at an irradiance of 150 mW/cm^2^. This time point was selected based on our previous study showing that Cet-PINs exhibited the highest level of specificity in 3D nodules at 6 h incubation time [65]. At 72 h following PDT treatment, the nodules were co-stained with LIVE (Calcein AM) and DEAD (propidium iodide) reagents prior to single-plane confocal imaging. For viability assessment of the 3D nodules in each experimental group, quantitative fractional viability heatmap images (Figure 7a,c) were generated using a comprehensive image analysis procedure for structurally complex organotypic cultures (CALYPSO) [81].

Untargeted-PSNs did not show any significant phototoxicity even at the highest concentration of 2000 nM of BPD-PC equivalent (Figure 7b,d). However, at a concentration of 2000 nM of BPD-PC equivalent, TR-PINs were most effective, and significantly reduced the viability of SCC-9 nodules to 45% and MIA PaCa-2 nodules to 24% (Figure 7b,d). Cet-PINs were equally effective at all concentrations of BPD-PC equivalent in the MIA PaCa-2 nodules and SCC-9 nodules.

MIA PaCa-2 nodules were more responsive to targeted PDT than the SCC-9 nodules at all concentrations of BPD-PC equivalent (Figure 7b,d). Importantly, in the absence of photoactivation, neither Cet-PINs nor TR-PINs exerted any toxic effects on cancer cells (Figure S2) at the concentration range of 0–2000 nM of BPD-PC equivalent, which is consistent with our previous findings using Cet-PINs [65].

**Figure 8:**
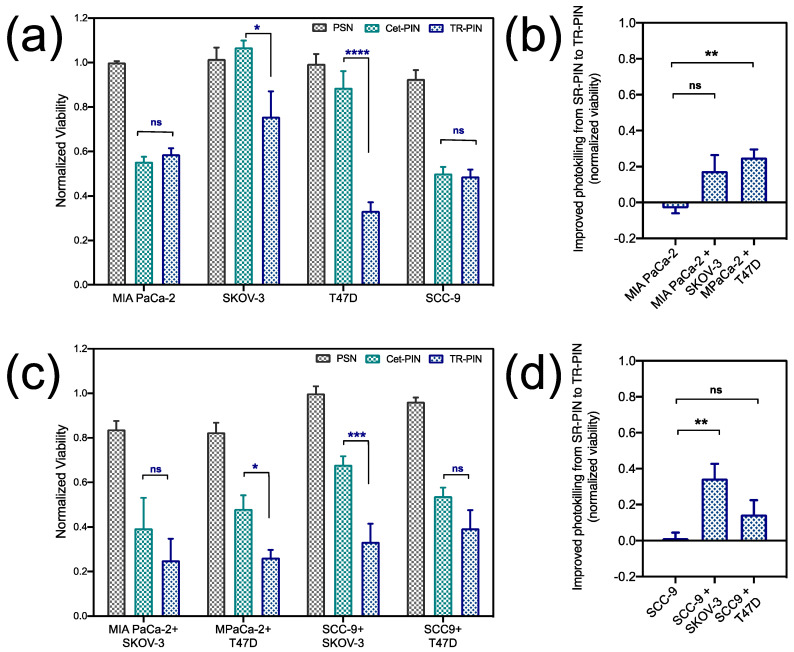
NIR light-mediated photodynamic treatment of 3D co-cultures of MIA PaCa-2 and SCC-9 cells with SKOV-3 and T47D cells. The CALYPSO image analysis framework was used for quantitation of normalized viability of (**a**) 3D monocultures and (**b**) co-cultures following PDT with untargeted-PSNs, Cet-targeted PINs (Cet-PINs), and triple-receptor-targeted TR-PINs, at a concentration of 500 nM of BPD-PC equivalent, (690 nm, 40 J/cm^2^ at 150 mW/cm^2^). (**c**,**d**) A comparison of normalized fractional viability of MIA PaCa-2 and SCC-9 monocultures and co-cultures with T47D and SKOV-3 following PDT with TR-PINs (690 nm, 40 J/cm^2^ at 150 mW/cm^2^). (mean ± S.E.M.; n = 8–12; one-way ANOVA with a Tukey post-test; **** = *p* ≤ 0.0001; *** = *p* ≤ 0.001; ** = *p* ≤ 0.01; * = *p* ≤ 0.05).

Triple-targeted PDT using TR-PINs was equally as effective as single-receptor EGFR-targeted Cet-PINs in the EGFR over-expressing MIA PaCa-2 and SCC-9 nodules. However, in the low-EGFR-expressing control nodules that over-express TfR (T47D) and HER-2 (SKOV-3), the TR-PINs were significantly more effective than the EGFR-targeted Cet-PINs (Figure 8a, Figure S3). The T47D nodule viability decreased by 67% after PDT with the TR-PINs (500 nM of BPD-PC equivalent), which was significantly more effective than the Cet-PINs. The SKOV-3 nodule viability decreased by 24% after PDT with the TR-PIN, whereas the Cet-PINs were ineffective at the same concentration (500 nM of BPD-PC equivalent). These T47D and SKOV-3 nodules represent low-EGFR-expressing tumors that would typically escape single-receptor EGFR-targeted PDT but would respond to the TR-PINs we report in this study.

As discussed earlier, tumors comprise of heterogeneous cells with various receptor expression profiles. Thus, even though a large proportion of tumor cells can be eradicated by single-receptor-targeted therapy, low-receptor-expressing subpopulations may persist and contribute to tumor recurrence. As such, we attempted to recapitulate heterogeneous PDAC and HNSCC tumors in vitro by forming heterocellular 3D models. MIA Paca-2 and SCC-9 nodules were formed with the addition of low-EGFR-expressing T47D or SKOV-3 cells that represent tumor subpopulations which we have shown to evade EGFR-targeted PDT using Cet-PINs (Figure 8a). While T47D and SKOV-3 cells express low levels of EGFR, treatment escape can be circumvented using TR-PINs by exploiting the over-expression of TfR in T47D cells and HER-2 in SKOV-3 cells. PDT treatment response in the heterogeneous heterocellular 3D nodules was then evaluated using Cet-PINs and TR-PINs (Figure 8c) and was compared with the treatment response in the monocellular 3D nodules. While the efficacy of TR-PINs was identical to that of the EGFR-targeted Cet-PINs in EGFR-over-expressing MIA PaCa-2 and SCC9 nodules, the TR-PINs were significantly more effective than the Cet-PINs in the heterogeneous heterocellular 3D nodules (Figure 8b,d). In heterocellular MIA PaCa-2 nodules containing SKOV-3 cells, TR-PINs provided a 17% greater reduction in viability than the Cet-PIN, and a 25% greater reduction in viability in heterocellular MIA PaCa-2 nodules containing T47D cells (Figure 7b). In heterocellular SCC9 nodules containing SKOV-3 cells, TR-PINs provided a 34% greater reduction in viability than the Cet-PIN, and a 14% greater reduction in viability in heterocellular MIA PaCa-2 nodules containing T47D cells (Figure 8d).

## 4. Discussion

Intratumoral heterogeneity can limit the efficacy of therapies directed towards single tumor cell surface receptors. Diverse patterns of tumor-associated cell surface receptor expression (EGFR, TfR, HER-2) have been found in PDAC and HNSCC patients. Specifically, studies have reported the over-expression of EGFR and HER-2 in patient pancreatic cancer tissue (45–95% and 43–69%, respectively) [60,97] and patient head and neck squamous cell carcinoma tissue (up to 90% and 68%, respectively) [25,98,99]. Thus, EGFR and HER-2 are both attractive targets for molecular targeted activatable therapies, such as PDT. However, one study using PDAC patient tissue found that HER-2 over-expression was concurrent with EGFR over-expression in 24% of patients, and in patients with no EGFR expression, no HER-2 over-expression was observed [100]. These findings emphasize that, in a clinical setting, targeting EGFR and HER-2 simultaneously using Cet-TZ-PINs may not be sufficient for effective and complete tumor eradication in all patients, and thus the TR-PINs we present here, with an expanded specificity for a third tumor receptors are critical. Considering that TfR over-expression has been found in PDAC [24] and HNSCC [32] and has also been found to play a prominent role in cancer cell proliferation, it is an important additional target for our TR-PIN-mediated PDT approach [44]. Thus, targeting three tumor receptors simultaneously may promote complete tumor eradication using spatiotemporally controlled activatable therapies such as PDT. Moreover, the emerging and promising use of bi-specific and multi-specific monoclonal antibodies (mAbs) directed towards multiple receptors is an additional motivation, which we further advance in the field by leveraging high-payload nanosystems [101,102]. In this study, we have developed NIR-activable triple-receptor-targeted photoimmuno-nanoconjugates (TR-PINs) with three ligands, conjugated to a single photosensitizing nanoconstruct to simultaneously target heterogeneous tumor cell subpopulations with differential expression levels of EGFR, TfR, and HER-2. These TR-PINs are proposed to increase the specificity and effective photodynamic eradication of tumor subpopulations in heterogeneous cancers with differential receptor expression levels.

We have assessed the cellular binding of the nanoconstructs by the quantitation of BPD-PC fluorescence emission using flow cytometry. Our results show that the cellular binding with TR-PINs was superior to both the Cet-PINs and Cet-TZ-PINs in tumor cells (A431, MIA PaCa-2, SCC-9, SKOV-3, T47D) with different origins and varying expression levels of tumor cell surface receptors. TR-PINs exhibit varying binding specificities to tumor cells (Table 3), thus the fold improvement in the cellular binding of TR-PINs (with respect to untargeted-PSN constructs) was found to be significantly higher than the calculated sum of the fold-improvements in binding with each PIN, with respect to untargeted-PSN constructs. This is likely due to the combined effect of the multiple ligands when conjugated on the surface of a single nanoconstruct to target multiple receptors simultaneously. This multiplicative increase in binding is likely due to multi-avidity effects, which can be advantageous; however, multi-target specificity remains to be the priority for targeting heterogeneity in this study.

The advantages of triple-receptor targeting are not only limited to enhanced surface binding to a greater proportion of heterogenous cancer cell subpopulations. This strategy also significantly increases the ability of PDAC and HNSCC tumor cells to internalize TR-PINs in vitro. PINs were found to localize to endo-lysosomal compartments when incubated with cells. Prior work using similar constructs also shows lysosomal localization of liposomal BPD-PC [94]. The staining with DAPI and lysotracker for confocal images does not show any membrane-bound constructs. Considering that, by 6 h, most of the PINs would have been internalized, as receptor-mediated endocytosis occurs from as early as 20 min following incubation [103], our prior work [65] corroborates the observations in this study whereby Cet-PINs were observed on the membrane of MIA PaCa-2 cells at 1 h incubation, but no membrane binding was observed in MIA PaCa-2 or OVCAR-5 cells at 6h following internalization. We also previously explored the relative binding and penetration of the Cet-PINs through 3D nodules [65]. Binding specificity of the Cet-PINs in 3D nodules of MIA PaCa-2 was quantified at different time points (1h, 6h, 24h). Cet-PINs exhibited the highest binding specificity (12.5-fold) for the MIA PaCa-2 3D nodules with respect to untargeted constructs at 6 h after incubation. In the current study, the fold improvement in binding with Cet-PINs is 23-fold compared with the untargeted-PSN in single-cell suspensions of MIA PaCa-2 cells. As such, it is apparent that the selectivity in uptake decreases from 2D to 3D cultures. These observations require further investigation before definitive and generalizable conclusions can be drawn.

We further evaluated the NIR phototoxicity in MIA PaCa-2 and SCC-9 nodules. TR-PINs are equally as effective as Cet-PINs in the MIA PaCa-2 and SSC-9 monocellular 3D nodules, even though the TR-PINs exhibit 1.7-fold and 1.4-fold higher cellular uptake, respectively. The absence of further improved PDT efficacy by triple targeting is likely to be a result of the 1.5-fold quenching of ^1^O_2_ in TR-PINs, as compared with Cet-PINs, when measured using DADB (Figure 6d). The results suggest that there is a minimum threshold in targeted cellular uptake after which the triple targeting will become more effective than the single targeting. These findings also suggest that improving the overall outcome of TR-PINs in heterogeneous tumors, as compared with Cet-PIN, may only become evident when the differences in the heterogeneous receptor expression exceed the 1.5-fold quenching of ^1^O_2_ observed.

As described earlier, tumors are heterogeneous masses of cancer cells with differential receptor expression profiles. Single-receptor-targeted therapies treat tumors with an assumption that they are a homogenous mass of cancer cells, and thus are generally only able to eradicate a specific proportion of tumor cells. The residual surviving tumor cells are thus likely to persist, proliferate aggressively, and promote tumor relapse and progression. Thus, the response to single-receptor-targeted therapies can be highly variable in these heterogeneous tumors. To address this heterogeneity-driven resistance to targeted therapies, we attempted to recapitulate heterogeneity in vitro, by forming 3D heterocellular models of MIA Paca-2 and SCC-9 cells, with the addition of low-EGFR-expressing T47D or SKOV-3 cells, that represent tumor subpopulations which we have shown to evade EGFR-targeted PDT using Cet-PINs (Figure 8a). While T47D and SKOV-3 cells express low levels of EGFR, treatment escape can be circumvented using TR-PINs by exploiting the over-expression of TfR in T47D cells and HER-2 in SKOV-3 cells. PDT treatment response in the heterogeneous heterocellular 3D nodules was then evaluated using Cet-PINs and TR-PINs (Figure 8c) and was compared with the treatment response in the monocellular 3D nodules. While the efficacy of TR-PINs was identical to that of the EGFR-targeted Cet-PINs in EGFR-over-expressing MIA PaCa-2 and SCC9 nodules, the TR-PINs were significantly more effective than the Cet-PINs in the heterogeneous heterocellular 3D nodules (Figure 8b,d). In heterocellular MIA PaCa-2 nodules containing SKOV-3 cells, TR-PINs provided a 17% greater reduction in viability than the Cet-PINs, and a 25% greater reduction in viability in heterocellular MIA PaCa-2 nodules containing T47D cells (Figure 8b). In heterocellular SCC9 nodules containing SKOV-3 cells, TR-PINs provided a 34% greater reduction in viability than the Cet-PINs, and a 14% greater reduction in viability in heterocellular MIA PaCa-2 nodules containing T47D cells (Figure 7d).

The significance of these findings is that they demonstrate that triple-receptor targeting using TR-PINs does provide more complete photodestruction of heterogeneous tumor nodules, which would otherwise partially evade single-receptor EGFR-targeted PDT. More complete PDT responses in heterogeneous tumors would thereby mitigate treatment escape, recurrence, and resistance to targeted therapy. Furthermore, TR-PINs would potentially be effective in a broader range of PDAC and HNSCC patients, in addition to patients with various other cancer indications where EGFR, HER-2, and TfR over-expression are implicated.

## 5. Conclusions

In this study, we show for the first time that heterogeneous heterocellular 3D models of PDAC and HNSCC can be more effectively destroyed using triple-receptor-targeted TR-PINs (EGFR-, HER-2-, and TfR-specific) that would otherwise partially evade single-receptor EGFR-targeted PDT. The significance of these findings specifically for PDT is that heterogeneous tumor subpopulations can also be effectively targeted using the TR-PINs, irrespective of the increase in cellular binding of the TR-PINs. PDT dosimetry can be modulated by tailoring the light dose applied, and thus, while cellular delivery is important, molecular specificity towards heterogeneous tumor cell subpopulations and discrimination between tumor tissue and healthy tissue remains critical.

Future work will further explore the encapsulation of multiple treatment modalities within a single TR-PIN construct. As such, heterogeneous tumor subpopulations would be simultaneously targeted with multiple treatment regimens that exhibit non-overlapping modes of cytotoxicity. Furthermore, PDT-based regimens using the TR-PIN platform will be evaluated in complex heterogeneous in vivo tumor models, such as patient-derived xenografts, to further mitigate the risk of treatment escape that often leads to tumor recurrence after an initial response.

## Figures and Tables

**Table 1:** Physical characterization of photoimmuno-nanoconjugates (PINs) and untargeted-photosensitizing nanoconstructs (untargeted-PSNs).

Nanoliposomes	Average Diameter (nm)	Polydispersity Index (PDI)	ζ-Potential (mV)	** Ligand Density on PINs
Cet-PIN	123.4 ± 0.2	0.05 ± 0.03	−16.7 ± 0.55	* 27.6 ± 1.6
Cet-TZ-PIN	144.1 ± 1.2	0.06 ± 0.01	−17.4 ± 1.04	36.3 ± 3.5
TR-PIN	126.9 ± 1.7	0.06 ± 0.00	−18.6 ± 1.01	89.6 ± 16.8
Untargeted-PSN	127.9 ± 1.3	0.08 ± 0.03	−18.5 ± 0.95	NA

** Ligand density (number of ligands/PIN) * values are mean ± S.D.

**Table 2:** Tumor-associated cell surface receptors (EGFR, HER-2, TfR) per cell.

Tumor Cell Lines	EGFR/Cell	TfR/Cell	HER-2/Cell
**A431**	2–4 × 10^6^ [82]	1.2 × 10^5^ [92]	1–2 × 10^5^ [91]
**T47D**	7.0 × 10^3^ [89]	NA	3 × 10^4^ [89]
**SKOV-3**	6.3 × 10^4^	5.6 × 10^5^	1.6 × 10^6^ [86]
**MIA PaCa-2**	1.7 × 10^5^ [93]	3.5 × 10^6^	6.7 × 10^4^
**SCC-9**	1.8 × 10^5^	3.2 × 10^6^	0.7 × 10^5^

**Table 3:** * Fold improvement in the cellular binding of nanoconstructs to the tumor cells.

Tumor Cell Lines	Fold Improvement with Cet-PINs	Fold Improvement with TZ-PINs	Fold Improvement with Cet-TZ-PINs	Fold Improvement with HT-PINs	Fold Improvement with TR-PINs
A431	24	1.7	57.1	4.8	111
T47D	1.5	1.7	3.8	8	9.2
SKOV-3	1.8	13.5	19.2	3.08	43.6
MIA PaCa-2	23.2	2.05	29	1.7	41.1
SCC-9	18.2	2.1	18	1.3	33

* (fold improvement in binding with targeting over untargeted-PSNs).

## Supplementary Materials

The following are available online at https://www.mdpi.com/2077-0383/9/8/2390/s1. Figure S1: Relative receptor expression of EGFR, HER-2, and TfR in SKOV-3 cells. Figure S2: Dark toxicity of untargeted-PSN, Cet-PINs, and TR-PINs in (a) MIA PaCa-2 and (b) SCC-9 cells. Figure S3: Normalized viability of (a) 3D monocellular and (b) heterocellular following PDT with untargeted-PSN, Cet-PINs, and TR-PIN, at a concentration of 250 nM of BPD-PC equivalent.

## References

[1] Celli J.P., Spring B.Q., Rizvi I., Evans C.L., Samkoe K.S., Verma S., Pogue B.W., Hasan T. (2010). Imaging and photodynamic therapy: Mechanisms, monitoring, and optimization. Chem. Rev..

[2] Baskaran R., Lee J., Yang S.-G. (2018). Clinical development of photodynamic agents and therapeutic applications. Biomater. Res..

[3] Fernandes S.R., Fernandes R., Sarmento B., Pereira P.M., Tomé J.P. (2019). Photoimmunoconjugates: Novel synthetic strategies to target and treat cancer by photodynamic therapy. Org. Biomol. Chem..

[4] Mew D., Wat C.-K., Towers G., Levy J. (1983). Photoimmunotherapy: Treatment of animal tumors with tumor-specific monoclonal antibody-hematoporphyrin conjugates. J. Immunol..

[5] Vrouenraets M.B., Visser G.W., Stewart F.A., Stigter M., Oppelaar H., Postmus P.E., Snow G.B., Van Dongen G.A. (1999). Development of meta-tetrahydroxyphenylchlorin-monoclonal antibody conjugates for photoimmunotherapy. Cancer Res..

[6] Hudson R., Carcenac M., Smith K., Madden L., Clarke O., Pelegrin A., Greenman J., Boyle R. (2005). The development and characterisation of porphyrin isothiocyanate–monoclonal antibody conjugates for photoimmunotherapy. Br. J. Cancer.

[7] Schmidt S., Wagner U., Oehr P., Krebs D. (1992). Clinical use of photodynamic therapy in gynecologic tumor patients–antibody-targeted photodynamic laser therapy as a new oncologic treatment procedure. Zent. Gynakol..

[8] Schmidt S., Wagner U., Schultes B., Oehr P., Decleer W., Ertmer W., Lubaschowski H., Biersack H., Krebs D. (1992). Photodynamic laser therapy with antibody-bound dyes. A new procedure in therapy of gynecologic malignancies. Fortschr. Med..

[9] Duska L.R., Hamblin M.R., Miller J.L., Hasan T. (1999). Combination photoimmunotherapy and cisplatin: Effects on human ovarian cancer ex vivo. J. Natl. Cancer. Inst..

[10] Soukos N.S., Hamblin M.R., Keel S., Fabian R.L., Deutsch T.F., Hasan T. (2001). Epidermal growth factor receptor-targeted immunophotodiagnosis and photoimmunotherapy of oral precancer in vivo. Cancer Res..

[11] Spring B.Q., Abu-Yousif A.O., Palanisami A., Rizvi I., Zheng X., Mai Z., Anbil S., Sears R.B., Mensah L.B., Goldschmidt R. (2014). Selective treatment and monitoring of disseminated cancer micrometastases in vivo using dual-function, activatable immunoconjugates. Proc. Natl. Acad. Sci. USA.

[12] Savellano M.D., Pogue B.W., Hoopes P.J., Vitetta E.S., Paulsen K.D. (2005). Multiepitope HER2 targeting enhances photoimmunotherapy of HER2-overexpressing cancer cells with pyropheophorbide-a immunoconjugates. Cancer Res..

[13] Del Governatore M., Hamblin M.R., Shea C.R., Rizvi I., Molpus K.G., Tanabe K.K., Hasan T. (2000). Experimental photoimmunotherapy of hepatic metastases of colorectal cancer with a 17.1 A chlorine6 immunoconjugate. Cancer Res..

[14] Molpus K.L., Hamblin M.R., Rizvi I., Hasan T. (2000). Intraperitoneal photoimmunotherapy of ovarian carcinoma xenografts in nude mice using charged photoimmunoconjugates. Gynecol. Oncol..

[15] Gillenwater A.M., Cognetti D., Johnson J.M., Curry J., Kochuparambil S.T., McDonald D., Fidler M.J., Stenson K., Vasan N., Razaq M. (2018). RM-1929 photo-immunotherapy in patients with recurrent head and neck cancer: Results of a multicenter phase 2a open-label clinical trial. Am. Soc. Clin. Oncol..

[16] Rybinski B., Yun K. (2016). Addressing intra-tumoral heterogeneity and therapy resistance. Oncotarget.

[17] Liu J., Dang H., Wang X.W. (2018). The significance of intertumor and intratumor heterogeneity in liver cancer. Exp. Mol. Med..

[18] Diaz-Cano S.J. (2012). Tumor heterogeneity: Mechanisms and bases for a reliable application of molecular marker design. Int. J. Mol. Sci..

[19] Huang M., Shen A., Ding J., Geng M. (2014). Molecularly targeted cancer therapy: Some lessons from the past decade. Trends Pharmacol. Sci..

[20] Alizadeh A.A., Aranda V., Bardelli A., Blanpain C., Bock C., Borowski C., Caldas C., Califano A., Doherty M., Elsner M. (2015). Toward understanding and exploiting tumor heterogeneity. Nat. Med..

[21] Pribluda A., Cecile C., Jackson E.L. (2015). Intratumoral heterogeneity: From diversity comes resistance. Clin. Cancer Res..

[22] Troiani T., Martinelli E., Capasso A., Morgillo F., Orditura M., De Vita F., Ciardiello F. (2012). Targeting EGFR in pancreatic cancer treatment. Curr. Drug Targets.

[23] Nedaeinia R., Avan A., Manian M., Salehi R., Ghayour-Mobarhan M. (2014). EGFR as a potential target for the treatment of pancreatic cancer: Dilemma and controversies. Curr. Drug Targets.

[24] Jeong S.M., Hwang S., Seong R.H. (2016). Transferrin receptor regulates pancreatic cancer growth by modulating mitochondrial respiration and ROS generation. Biochem. Biophys. Res. Commun..

[25] Pollock N.I., Grandis J.R. (2015). HER2 as a therapeutic target in head and neck squamous cell carcinoma. Clin. Cancer Res..

[26] Del Campo J., Hitt R., Sebastian P., Carracedo C., Lokanatha D., Bourhis J., Temam S., Cupissol D., De Raucourt D., Maroudias N. (2011). Effects of lapatinib monotherapy: Results of a randomised phase II study in therapy-naive patients with locally advanced squamous cell carcinoma of the head and neck. Br. J. Cancer.

[27] Williams M.D., Roberts D.B., Kies M.S., Mao L., Weber R.S., El-Naggar A.K. (2010). Genetic and expression analysis of HER-2 and EGFR genes in salivary duct carcinoma: Empirical and therapeutic significance. Clin. Cancer Res..

[28] Nardi V., Sadow P.M., Juric D., Zhao D., Cosper A.K., Bergethon K., Scialabba V.L., Batten J.M., Borger D.R., Iafrate A.J. (2013). Detection of novel actionable genetic changes in salivary duct carcinoma helps direct patient treatment. Clin. Cancer Res..

[29] Hendler F., Ozanne B. (1984). Human squamous cell lung cancers express increased epidermal growth factor receptors. J. Clin. Investig..

[30] Hanken H., Gaudin R., Gröbe A., Fraederich M., Eichhorn W., Smeets R., Simon R., Sauter G., Grupp K., Izbicki J.R. (2014). Her2 expression and gene amplification is rarely detectable in patients with oral squamous cell carcinomas. J. Oral Pathol. Med..

[31] Falchook G.S., Lippman S.M., Bastida C.C., Kurzrock R. (2014). Human epidermal receptor 2-amplified salivary duct carcinoma: Regression with dual human epidermal receptor 2 inhibition and anti-vascular endothelial growth factor combination treatment. Head Neck.

[32] Kearsley J., Furlong K., Cooke R., Waters M. (1990). An immunohistochemical assessment of cellular proliferation markers in head and neck squamous cell cancers. Br. J. Cancer.

[33] Masuda H., Zhang D., Bartholomeusz C., Doihara H., Hortobagyi G.N., Ueno N.T. (2012). Role of epidermal growth factor receptor in breast cancer. Breast Cancer Res. Treat..

[34] Iqbal N., Iqbal N. (2014). Human epidermal growth factor receptor 2 (HER2) in cancers: Overexpression and therapeutic implications. Mol. Biol. Int..

[35] Rychtarcikova Z., Lettlova S., Tomkova V., Korenkova V., Langerova L., Simonova E., Zjablovskaja P., Alberich-Jorda M., Neuzil J., Truksa J. (2017). Tumor-initiating cells of breast and prostate origin show alterations in the expression of genes related to iron metabolism. Oncotarget.

[36] Teplinsky E., Muggia F. (2015). EGFR and HER2: Is there a role in ovarian cancer?. Transl. Cancer Res..

[37] Basuli D., Tesfay L., Deng Z., Paul B., Yamamoto Y., Ning G., Xian W., McKeon F., Lynch M., Crum C.P. (2017). Iron addiction: A novel therapeutic target in ovarian cancer. Oncogene.

[38] Scagliotti G.V., Selvaggi G., Novello S., Hirsch F.R. (2004). The biology of epidermal growth factor receptor in lung cancer. Clin. Cancer Res..

[39] Yan M., Parker B.A., Schwab R., Kurzrock R. (2014). HER2 aberrations in cancer: Implications for therapy. Cancer Treat. Rev..

[40] Wang B., Zhang J., Song F., Tian M., Shi B., Jiang H., Xu W., Wang H., Zhou M., Pan X. (2016). EGFR regulates iron homeostasis to promote cancer growth through redistribution of transferrin receptor 1. Cancer Lett..

[41] Zhu X., Zhang H., Lin Y., Chen P., Min J., Wang Z., Xiao W., Chen B. (2009). Mechanisms of gambogic acid-induced apoptosis in non-small cell lung cancer cells in relation to transferrin receptors. J. Chemother..

[42] Chaux A., Cohen J.S., Schultz L., Albadine R., Jadallah S., Murphy K.M., Sharma R., Schoenberg M.P., Netto G.J. (2012). High epidermal growth factor receptor immunohistochemical expression in urothelial carcinoma of the bladder is not associated with EGFR mutations in exons 19 and 21: A study using formalin-fixed, paraffin-embedded archival tissues. Hum. Pathol..

[43] Rahman S.A., Yokoyama M., Nishio S., Takeuchi M. (1997). Flow cytometric evaluation of transferrin receptor in transitional cell carcinoma. Urol. Res..

[44] Baselga J., Swain S.M. (2009). Novel anticancer targets: Revisiting ERBB2 and discovering ERBB3. Nat. Rev. Cancer.

[45] Seshacharyulu P., Ponnusamy M.P., Haridas D., Jain M., Ganti A.K., Batra S.K. (2012). Targeting the EGFR signaling pathway in cancer therapy. Expert Opin. Ther. Targets.

[46] Tebbutt N., Pedersen M.W., Johns T.G. (2013). Targeting the ERBB family in cancer: Couples therapy. Nat. Rev. Cancer.

[47] Kol A., van Scheltinga A.G.T., Timmer-Bosscha H., Lamberts L.E., Bensch F., de Vries E.G., Schröder C.P. (2014). HER3, serious partner in crime: Therapeutic approaches and potential biomarkers for effect of HER3-targeting. Pharmacol. Ther..

[48] Szekeres T., Sedlak J., Novotny L. (2002). Benzamide riboside, a recent inhibitor of inosine 5′-monophosphate dehydrogenase induces transferrin receptors in cancer cells. Curr. Med. Chem..

[49] Ryschich E., Huszty G., Knaebel H., Hartel M., Büchler M., Schmidt J. (2004). Transferrin receptor is a marker of malignant phenotype in human pancreatic cancer and in neuroendocrine carcinoma of the pancreas. Eur. J. Cancer.

[50] Daniels-Wells T.R., Penichet M.L. (2016). Transferrin receptor 1: A target for antibody-mediated cancer therapy. Immunotherapy.

[51] García-Foncillas J., Sunakawa Y., Aderka D., Wainberg Z., Ronga P., Witzler P., Stintzing S. (2019). Distinguishing Features of Cetuximab and Panitumumab in Colorectal Cancer and Other Solid Tumors. Front. Oncol..

[52] Vermorken J.B., Mesia R., Rivera F., Remenar E., Kawecki A., Rottey S., Erfan J., Zabolotnyy D., Kienzer H.-R., Cupissol D. (2008). Platinum-based chemotherapy plus cetuximab in head and neck cancer. N. Engl. J. Med..

[53] Thatcher N., Hirsch F.R., Luft A.V., Szczesna A., Ciuleanu T.E., Dediu M., Ramlau R., Galiulin R.K., Bálint B., Losonczy G. (2015). Necitumumab plus gemcitabine and cisplatin versus gemcitabine and cisplatin alone as first-line therapy in patients with stage IV squamous non-small-cell lung cancer (SQUIRE): An open-label, randomised, controlled phase 3 trial. Lancet Oncol..

[54] Maximiano S., Magalhaes P., Guerreiro M.P., Morgado M. (2016). Trastuzumab in the Treatment of Breast Cancer. BioDrugs.

[55] Ross J.S., Mulcahy M. (2011). HER2 Testing in Gastric/Gastroesophageal Junction Adenocarcinomas: Unique Features of a Familiar Test. Gastrointest. Cancer Res..

[56] Daniels T.R., Bernabeu E., Rodríguez J.A., Patel S., Kozman M., Chiappetta D.A., Holler E., Ljubimova J.Y., Helguera G., Penichet M.L. (2012). The transferrin receptor and the targeted delivery of therapeutic agents against cancer. Biochim. Biophys Acta.

[57] Jiang N., Wang D., Hu Z., Shin H.J., Qian G., Rahman M.A., Zhang H., Amin A.R., Nannapaneni S., Wang X. (2014). Combination of anti-HER3 antibody MM-121/SAR256212 and cetuximab inhibits tumor growth in preclinical models of head and neck squamous cell carcinoma. Mol. Cancer Ther..

[58] Vermorken J.B., Trigo J., Hitt R., Koralewski P., Diaz-Rubio E., Rolland F., Knecht R., Amellal N., Schueler A., Baselga J. (2007). Open-label, uncontrolled, multicenter phase II study to evaluate the efficacy and toxicity of cetuximab as a single agent in patients with recurrent and/or metastatic squamous cell carcinoma of the head and neck who failed to respond to platinum-based therapy. J. Clin. Oncol..

[59] Saxby A.J., Nielsen A., Scarlett C.J., Clarkson A., Morey A., Gill A., Smith R.C. (2005). Assessment of HER-2 status in pancreatic adenocarcinoma: Correlation of immunohistochemistry, quantitative real-time RT-PCR, and FISH with aneuploidy and survival. Am. J. Surg. Pathol..

[60] Larbouret C., Robert B., Navarro-Teulon I., Thèzenas S., Ladjemi M.-Z., Morisseau S., Campigna E., Bibeau F., Mach J.-P., Pèlegrin A. (2007). In vivo therapeutic synergism of anti–epidermal growth factor receptor and anti-HER2 monoclonal antibodies against pancreatic carcinomas. Clin. Cancer Res..

[61] Day J.D., Digiuseppe J.A., Yeo C., Lai-Goldman M., Anderson S.M., Goodman S.N., Kern S.E., Hruban R.H. (1996). Immunohistochemical evaluation of HER-2/neu expression in pancreatic adenocarcinoma and pancreatic intraepithelial neoplasms. Hum. Pathol..

[62] von Roemeling C., Jiang W., Chan C.K., Weissman I.L., Kim B.Y. (2017). Breaking down the barriers to precision cancer nanomedicine. Trends Biotechnol..

[63] Obaid G., Broekgaarden M., Bulin A.-L., Huang H.-C., Kuriakose J., Liu J., Hasan T. (2016). Photonanomedicine: A convergence of photodynamic therapy and nanotechnology. Nanoscale.

[64] Lucky S.S., Soo K.C., Zhang Y. (2015). Nanoparticles in photodynamic therapy. Chem. Rev..

[65] Obaid G., Bano S., Mallidi S., Broekgaarden M., Kuriakose J., Silber Z., Bulin A.L., Wang Y., Mai Z., Jin W. (2019). Impacting Pancreatic Cancer Therapy in Heterotypic in Vitro Organoids and in Vivo Tumors with Specificity-Tuned, NIR-Activable Photoimmunonanoconjugates: Towards Conquering Desmoplasia?. Nano Lett..

[66] Stefanick J.F., Omstead D.T., Kiziltepe T., Bilgicer B. (2019). Dual-receptor targeted strategy in nanoparticle design achieves tumor cell selectivity through cooperativity. Nanoscale.

[67] Dixit S., Miller K., Zhu Y., McKinnon E., Novak T., Kenney M.E., Broome A.-M. (2015). Dual Receptor-Targeted Theranostic Nanoparticles for Localized Delivery and Activation of Photodynamic Therapy Drug in Glioblastomas. Mol. Pharm..

[68] Bhattacharyya S., Khan J.A., Curran G.L., Robertson J.D., Bhattacharya R., Mukherjee P. (2011). Efficient delivery of gold nanoparticles by dual receptor targeting. Adv. Mater..

[69] Li Q., Li W., Di H., Luo L., Zhu C., Yang J., Yin X., Yin H., Gao J., Du Y. (2018). A photosensitive liposome with NIR light triggered doxorubicin release as a combined photodynamic-chemo therapy system. J. Control. Release.

[70] Sneider A., Jadia R., Piel B., VanDyke D., Tsiros C., Rai P. (2017). Engineering Remotely Triggered Liposomes to Target Triple Negative Breast Cancer. Oncomedicine.

[71] Paszko E., Vaz G.M., Ehrhardt C., Senge M.O. (2013). Transferrin conjugation does not increase the efficiency of liposomal Foscan during in vitro photodynamic therapy of oesophageal cancer. Eur. J. Pharm. Sci..

[72] Guo P., Yang J., Liu D., Huang L., Fell G., Huang J., Moses M.A., Auguste D.T. (2019). Dual complementary liposomes inhibit triple-negative breast tumor progression and metastasis. Sci. Adv..

[73] Kontermann R.E. (2012). Dual targeting strategies with bispecific antibodies. MAbs.

[74] Gao J.-Q., Lv Q., Li L.-M., Tang X.-J., Li F.-Z., Hu Y.-L., Han M. (2013). Glioma targeting and blood–brain barrier penetration by dual-targeting doxorubincin liposomes. Biomaterials.

[75] Eniola A.O., Hammer D.A. (2005). In vitro characterization of leukocyte mimetic for targeting therapeutics to the endothelium using two receptors. Biomaterials.

[76] Saul J.M., Annapragada A.V., Bellamkonda R.V. (2006). A dual-ligand approach for enhancing targeting selectivity of therapeutic nanocarriers. J. Control. Release.

[77] Vaidya T., Straubinger R.M., Ait-Oudhia S. (2018). Development and evaluation of tri-functional immunoliposomes for the treatment of HER2 positive breast cancer. Pharm. Res..

[78] Lörz M., Meyer-Breiting E. (1991). Transferrin receptors in squamous epithelial cancers of the head and neck. Laryngo-Rhino-Otol..

[79] Heitner T., Moor A., Garrison J.L., Marks C., Hasan T., Marks J.D. (2001). Selection of cell binding and internalizing epidermal growth factor receptor antibodies from a phage display library. J. Immunol. Methods.

[80] Obaid G., Jin W., Bano S., Kessel D., Hasan T. (2019). Nanolipid Formulations of Benzoporphyrin Derivative: Exploring the Dependence of Nanoconstruct Photophysics and Photochemistry on Their Therapeutic Index in Ovarian Cancer Cells. Photochem. Photobiol..

[81] Bulin A.L., Broekgaarden M., Hasan T. (2017). Comprehensive high-throughput image analysis for therapeutic efficacy of architecturally complex heterotypic organoids. Sci. Rep..

[82] Gadella T., Jovin T.M. (1995). Oligomerization of epidermal growth factor receptors on A431 cells studied by time-resolved fluorescence imaging microscopy. A stereochemical model for tyrosine kinase receptor activation. J. Cell Biol..

[83] Abe K., Zhao L., Periasamy A., Intes X., Barroso M. (2013). Non-invasive in vivo imaging of near infrared-labeled transferrin in breast cancer cells and tumors using fluorescence lifetime FRET. PLoS ONE.

[84] Shailaja K., Katayoun A.J., Sergei M., Jean Yu W., Nicole M.E., John D., Ling Q., Shannon P.A., Anthony E.P., Nilantha S. (2005). A Role for Transferrin Receptor in Triggering Apoptosis When Targeted with Gambogic Acid. Proc. Natl. Acad. Sci. USA.

[85] Bijman M.N., van Berkel M.P., Kok M., Janmaat M.L., Boven E. (2009). Inhibition of functional HER family members increases the sensitivity to docetaxel in human ovarian cancer cell lines. Anti-Cancer Drugs.

[86] Tolmachev V., Tran T.A., Rosik D., Sjöberg A., Abrahmsén L., Orlova A. (2012). Tumor targeting using affibody molecules: Interplay of affinity, target expression level, and binding site composition. J. Nucl. Med..

[87] Abu-Yousif A.O., Moor A.C., Zheng X., Savellano M.D., Yu W., Selbo P.K., Hasan T. (2012). Epidermal growth factor receptor-targeted photosensitizer selectively inhibits EGFR signaling and induces targeted phototoxicity in ovarian cancer cells. Cancer Lett..

[88] Rutledge E.A., Green F.A., Enns C.A. (1994). Generation of the soluble transferrin receptor requires cycling through an endosomal compartment. J. Biol. Chem..

[89] Beerli R.R., Graus-Porta D., Woods-Cook K., Chen X., Yarden Y., Hynes N.E. (1995). Neu differentiation factor activation of ErbB-3 and ErbB-4 is cell specific and displays a differential requirement for ErbB-2. Mol. Cell. Biol..

[90] Modjtahedi H., Komurasaki T., Toyoda H., Dean C. (1998). Anti-EGFR monoclonal antibodies which act as EGF, TGFα, HB-EGF and BTC antagonists block the binding of epiregulin to EGFR-expressing tumours. Int. J. Cancer.

[91] Bjorkelund H., Gedda L., Barta P., Malmqvist M., Andersson K. (2011). Gefitinib Induces Epidermal Growth Factor Receptor Dimers Which Alters the Interaction Characteristics with. sup. 125I-EGF. PLoS ONE.

[92] Hopkins C.R., Trowbridge I.S. (1983). Internalization and processing of transferrin and the transferrin receptor in human carcinoma A431 cells. J. Cell Biol..

[93] Smith J.J., Derynck R., Korc M. (1987). Production of transforming growth factor alpha in human pancreatic cancer cells: Evidence for a superagonist autocrine cycle. Proc. Natl. Acad. Sci. USA.

[94] Rizvi I., Obaid G., Bano S., Hasan T., Kessel D. (2018). Photodynamic therapy: Promoting in vitro efficacy of photodynamic therapy by liposomal formulations of a photosensitizing agent. Lasers Surg. Med..

[95] Kessel D., Price M. (2012). Evaluation of Diethyl-3-3′-(9,10-anthracenediyl) bis Acrylate as a Probe for Singlet Oxygen Formation during Photodynamic Therapy. Photochem. Photobiol..

[96] Michaeli A., Feitelson J. (1994). Reactivity of singlet oxygen toward amino acids and peptides. Photochem. Photobiol..

[97] Bossi P., Resteghini C., Paielli N., Licitra L., Pilotti S., Perrone F. (2016). Prognostic and predictive value of EGFR in head and neck squamous cell carcinoma. Oncotarget.

[98] Khademi B., Shirazi F.M., Vasei M., Doroudchi M., Gandomi B., Modjtahedi H., Pezeshki A.M., Ghaderi A. (2002). The expression of p53, c-erbB-1 and c-erbB-2 molecules and their correlation with prognostic markers in patients with head and neck tumors. Cancer Lett..

[99] Azemar M., Schmidt M., Arlt F., Kennel P., Brandt B., Papadimitriou A., Groner B., Wels W. (2000). Recombinant antibody toxins specific for ErbB2 and EGF receptor inhibit the in vitro growth of human head and neck cancer cells and cause rapid tumor regression in vivo. Int. J. Cancer.

[100] Dancer J., Takei H., Ro J.Y., Lowery-Nordberg M. (2007). Coexpression of EGFR and HER-2 in pancreatic ductal adenocarcinoma: A comparative study using immunohistochemistry correlated with gene amplification by fluorescencent in situ hybridization. Oncol. Rep..

[101] Wu L., Seung E., Xu L., Rao E., Lord D.M., Wei R.R., Cortez-Retamozo V., Ospina B., Posternak V., Ulinski G. (2020). Trispecific antibodies enhance the therapeutic efficacy of tumor-directed T cells through T cell receptor co-stimulation. Nat. Cancer.

[102] Runcie K., Budman D.R., John V., Seetharamu N. (2018). Bi-specific and tri-specific antibodies-the next big thing in solid tumor therapeutics. Mol. Med..

[103] Gao H., Shi W., Freund L.B. (2005). Mechanics of receptor-mediated endocytosis. Proc. Natl. Acad. Sci. USA.

