# Supplementary material for: NIR Photodynamic Destruction of PDAC and HNSCC Nodules Using Triple-Receptor-Targeted Photoimmuno-Nanoconjugates: Targeting Heterogeneity in Cancer
Source: J Clin Med. 2020 Jul 27;9(8):2390. doi: 10.3390/jcm9082390 (PMC7464411; doi:10.3390/jcm9082390)
Supplement: Supplementary file 1 [file jcm-09-02390-s001.pdf]

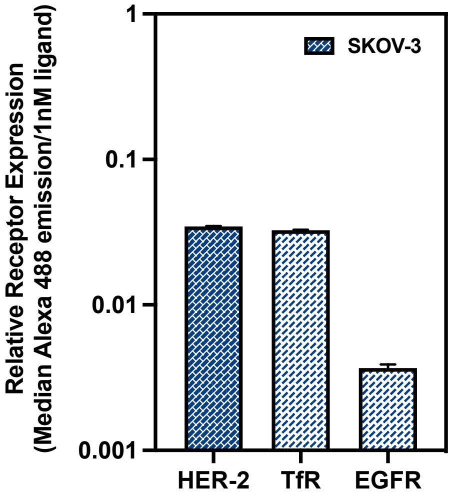

Figure S1. Relative receptor expression of EGFR, HER-2 and TfR in SKOV-3 cells determined using flow cytometry and represented as median fluorescence emission of Alexa Fluor 488, when conjugated to either cetuximab, transferrin or trastuzumab respectively. (mean ± S.E.M.; *n* = 3).

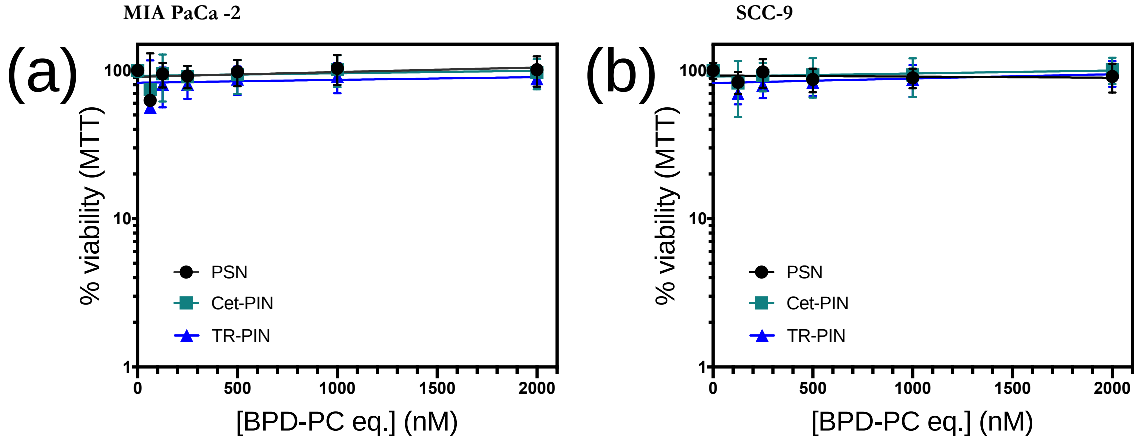

Figure S2. *In vitro* dark toxicity of untargeted-PSN, Cet-PIN and TR-PIN in (a) MIA PaCa-2 and (b) SCC-9 (c) cells. (mean ± S.E.M.; *n* = 8–12).

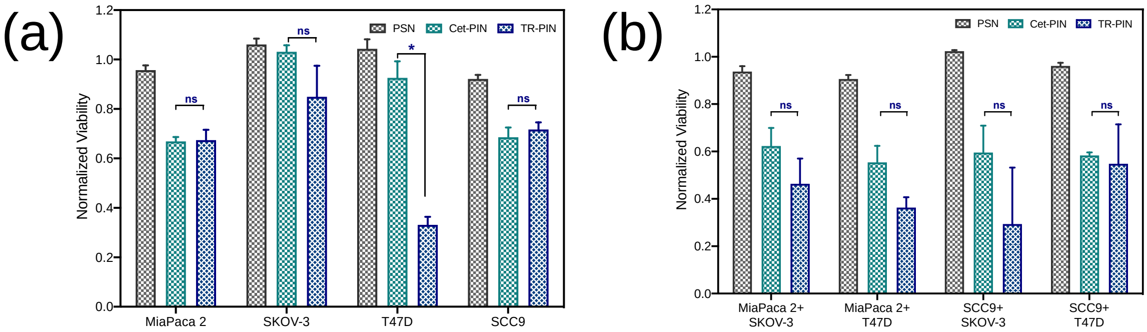

Figure S3. Normalized viability of (a) 3D monocellular and (b) heterocellular nodules, following PDT with untargeted-PSN, Cet-PIN and TR-PIN, at a concentration of 250 nM of BPD-PC equivalent, (690 nm, 40J/cm^2 at 150 mW/cm^2). (mean ± S.E.M.; *n* = 8–12; One-Way ANOVA with a Tukey Post-Test; *∗ = P ≤ 0.05).*
